# Helminth-induced prostaglandin signaling and dietary shifts in PUFA metabolism promote colitis-associated cancer

**DOI:** 10.1016/j.jlr.2025.100837

**Published:** 2025-06-07

**Authors:** Katherine A. Smith, Ella K. Reed, Irina Guschina, Victoria J. Tyrrell, Claire Butters, Matthew G. Darby, Brunette Katsandegwaza, Alisha Chetty, William G.C. Horsnell, Valerie B. O’Donnell, Awen Gallimore

**Affiliations:** 1 Cardiff University, School of Biosciences, Cardiff, UK; 2 Cardiff University, School of Medicine, Cardiff, UK; 3 University of Cape Town, Institute of Infectious Disease and Molecular Medicine, Cape Town, South Africa; 4 Department of Infectious and Parasite Diseases, University of Liege, Liege, Belgium; 5 University of Exeter, Medical Research Council Centre for Medical Mycology, Exeter, UK

**Keywords:** arachidonic acid, cell signaling, colon, lipolysis and fatty acid metabolism, lipoxygenase, omega-3 fatty acids, phospholipids/metabolism, prostaglandins

## Abstract

Oxylipins derived from dietary polyunsaturated fatty acids (PUFAs) are key determinants of intestinal health, homeostasis, and inflammatory disorders, such as colitis-associated colorectal cancer. Previous research has independently linked a high dietary omega (ω)-6:ω-3 PUFA ratio, or intestinal helminth infection, to an increased risk of colitis-associated colorectal cancer. However, whether these two factors interact to exacerbate disease risk and whether oxylipins contribute to this is unknown. In this study, we report that infection with the helminth *Heligmosomoides polygyrus bakeri* (Hpb) exacerbates tumor formation when combined with a high ω-6:ω-3 PUFA ratio diet. Dietary increases in tumor burden correlated with heightened levels of arachidonic acid (AA) and AA-derived lipoxygenase (LOX) oxylipins in the colon, including the 12/15-LOX product 12-hydroxyeicosatetraenoic acid, prior to disease onset. Although helminth infection further increased the production of 12/15-LOX oxylipins and increased expression of *Alox15*, responsible for producing these metabolites, inhibition of cyclooxygenase-dependent prostaglandin production with aspirin prevented helminth-exacerbation of disease. Helminth-infected mice exhibited increased phosphorylation of β-catenin in the colon, which was inhibited by EP2 and 4 antagonists. Moreover, administration of an EP agonist increased tumor burden in naive mice fed a high ω-6:ω-3 PUFA ratio diet, to the levels seen in helminth-exacerbation of disease. These data suggest that dietary changes in fatty acid composition coordinate with helminth-induced activation of EP signaling to exacerbate tumor development.

Colorectal cancer is the third most common cancer and the second leading cause of cancer-related death worldwide. In 2020, countries with the lowest human development index (HDI), which measures social and economic development, exhibited the lowest incidence of this disease compared to those with a very high HDI. However, projections for 2040 estimate a significant 103% increase in cases in low HDI countries, compared to a more moderate 35% increase anticipated in very high HDI countries (1). Many factors are thought to influence colorectal cancer development, including genetic risk as well as lifestyle choices. Patients with chronic inflammatory bowel disease (IBD) colitis are at an increased risk of developing colitis-associated colorectal cancer (CAC) (2, 3, 4), reflecting shared molecular mechanisms underlying these diseases.

Changes in diet are suggested to significantly impact colorectal cancer incidence (5, 6). Notably, its rise in low HDI countries has been linked with urbanization, and the adoption of “Western” diets (7, 8), characterized by high ratios of ω-6:ω-3 polyunsaturated fatty acid (PUFA) that are now estimated to range between 20:1 and 50:1 (9). Diets rich in linoleic acid (LA), an omega-6 (ω-6) PUFA, which is converted into arachidonic acid (AA) in vivo, are associated with increased IBD and colorectal cancer in humans and mouse models of disease (10, 11, 12, 13, 14). Murine studies have demonstrated a role for AA-derived oxylipins generated by cyclooxygenase (COX) in CAC using pharmacological tools such as aspirin, which also significantly reduces the risk of colorectal cancer incidence and improves disease-associated survival in humans (15, 16). However, high levels of oxylipins derived from AA by lipoxygenases (LOXs) were reported in patient colon adenoma tissue samples (17) and several preclinical models have described the importance of 12/15-LOX-derived oxylipins, including 12-hydroxyeicosatetranoic acid (HETE), in promoting colorectal cancer cell growth and invasion (18, 19, 20). In line with the idea of shared mechanisms underlying disease in CAC and IBD, a significant reduction in dextran sulfate sodium (DSS) colitis in *Alox15*-deficient mice was linked to decreased production of 12-HETE, 15-HETE, and 5-HETE (21).

Separate from diet, growing evidence indicates a positive association between helminth infection and colorectal cancer in humans and murine models (22, 23, 24, 25). Soil-transmitted helminths (STH) are highly prevalent in low HDI countries, affecting an estimated 1.45 billion people worldwide (26). Recent studies in mice indicate that rodent STH transit through tissue can trigger COX and LOX-dependent oxylipin production from AA at that site following infection (27). In addition, a glutamate dehydrogenase enzyme found in the infectious larval products of the rodent STH *Heligmosomoides polygyrus bakeri* (Hpb) and larval cysts of the zoonotic tapeworm *Taenia Solium* promoted prostaglandin E_2_ (PGE_2_) production from macrophages and monocytes (28, 29). It is currently not known whether the oxylipins generated following helminth exposure contribute to colorectal cancer risk or progression of disease.

Mapping an increasing adoption of high ω-6:ω-3 ratio diets onto regions with high helminth infection rates, the question arises as to whether the combination of these factors further influences cancer incidence, and if so, whether oxylipins play a role. This is crucial to determine now, as helminth-endemic areas are reported to be undergoing a rapid nutritional transition to diets with a higher ω-6:ω-3 ratio (30, 31). To address whether both risk factors exacerbate disease by targeting the same oxylipin pathways, we tested the impact of a high ω-6:ω-3 ratio diet on tumor formation and oxylipin generation in mice in a model of DSS CAC, with or without concurrent helminth infection. The contribution of oxylipins generated by COX to driving CAC in this model is elucidated along with signaling mechanisms. Our data reveal two independent oxylipin pathways that are associated with an increased risk of colorectal cancer in mice: one that can be modified by diet and the other by the delivery of aspirin during helminth infection.

## Materials and Methods

### Experimental model details

Female 6-8-week-old mice were bred and maintained in-house under specific pathogen-free level 1-barrier conditions within a South African Veterinary Council authorized facility at the University of Cape Town (registration number FR15/14226). Serum and fecal screening revealed mouse norovirus, *Pasterella pneumotropica*, *Helicobacter spp*., and *Tritrichomonas muris*. Based on 369 of the 384 SNP loci, sequence genotyping of a tail snip from this strain by Charles River revealed a 90.2% match with the BALB/cByJ reference genome and an average 5.8% homozygous mismatch to this strain, including a 47.6% match to 129S4SvJae instead of a 45.2% match found for the reference BALBcByJ strain. Littermates were randomly assigned to experimental groups, housed within the same cage. Mice received standard laboratory chow containing low levels of ω-6 (Rodent Breeder, Cat. #RB2005, LabChef, Nutritionhub) and drinking water from weening ad libitum. CAC was promoted in this strain using a proinflammatory high sucrose American Institute of Nutrition (AIN)-76A rodent diet (32). Animals were switched to an unmodified AIN-76A diet containing an ω-6:ω-3 ratio of 43:1 (Product #D10001, Research Diets), a modified AIN-76A diet with a high ω-6:ω-3 ratio of 120:1 (Product #D16083101, Research Diets), or maintained on standard laboratory chow for one week before helminth infection or CAC induction. The composition of each diet is shown, where NG = not given by manufacturer (Table 1). Mice were then maintained in each diet until euthanasia. All protocols were approved by the University of Cape Town animal ethics committee (AEC 015/001, 019/010) and following South African Veterinary Council authorization (AR15/13922).

**Table 1:** Composition of rodent diets

Diet	Standard Laboratory Chow		AIN-76A		Modified AIN-76A	
Product #	2005		D10001		D16083101	
Component	g/kg	g%	g/kg	g%	g/kg	g%
Protein	220	22 (soybean, maize protein concentrate, fishmeal)	200	20 (casein)	200	20 (casein)
Carbohydrate	520	52 (maize, wheat bran, soybean, sucrose)	660	66 (corn starch, sucrose, cellulose)	660	66 (corn starch, sucrose, cellulose)
Fiber	40	4 (soybean)	50	5 (all cellulose)	50	5 (all cellulose)
Fat	50	5 (maize, soybean, wheat bran)	50	5 (corn oil)	50	5 (sunflower oil, high linoleic)
Linoleic acid (ω-6)	12	1.2	30.05	3.005	30.1	3.01
α-linolenic acid (ω-3)	NG	NG	0.7	0.07	0.25	0.025
Sucrose	40–60	4–6	500	50	500	50
ω-6:ω-3 ratio	NG		42.9:1		120.4:1	

### CAC induction

Azoxymethane (AOM) (Merck A5486) was delivered intraperitoneally (i.p.) at a dose of 8–12.5 mg/kg followed by three fortnightly 5–7-day cycles of 2–2.5% 40,000–50,000 MW DSS (Affymetrix/USB J14489) in the water. 16, 16-dimethyl prostaglandin E_2_ (dmePGE2) was administered i.p. to BALB/c mice at a dose of 12 μg/kg and control mice received 200 μl 1:1 DMSO:PBS. Low-dose aspirin was administered in the drinking water at a dose of 25 mg/kg/day, given fresh every day. EP2 and EP4 antagonists PF-04418948 and ONO-AE3-208 were given i.p. at a dose of 10 mg/kg at day −10, −7, −4, and −1 before AOM administration. 1:1 DMSO:PBS was given i.p. as a vehicle control. Mice were monitored daily for weight loss, physical, and behavioral changes and were immediately euthanized if the humane end point was reached. For tumor burden quantification, the entire colon was removed from below the cecum to the anus, measured, and flushed with PBS and opened longitudinally before fixation in 4% paraformaldehyde. The colon was then pinned out before enumerating tumor burden macroscopically by an observer blinded to treatment group.

### Helminth infection

*H. polygyrus (bakeri)* (Hpb) was maintained as described elsewhere (33). Mice were infected with 200 Hpb L3 larvae using a 21G gavage needle in 200 μl of sterile water.

### Oxylipin analysis

Subsequently, 0.5 cm tissue samples taken from the distal colon were snap frozen on dry ice, weighed, and added to ceramic beads in 1 ml antioxidant buffer containing 100 μM diethylenetriaminepentaacetic acid and 100 μM butylated hydroxytoluene in phosphate buffered saline, as well as 2.1–2.9 ng of 13(S)-HODE-d4, 5(S)-HETE-d8, 12(S)-HETE-d8, 15(S)-HETE-d8, 20-HETE-d6, LTB_4_-d4, Resolvin D1-d5, PGE_2_-d4, PGD_2_-d4, PGF_2_α-d4, TXB_2_-d4, and 11-dehydro-thromboxane B2-d4 standard (Cayman chemicals). They were homogenized using a Bead Ruptor Elite for 2 × 20-s intervals at six m/s under cooled nitrogen gas (4°C). Lipids were extracted by adding a 2.5 ml solvent mixture (1 M acetic acid/isopropanol/hexane; 2:20:30, v/v/v) to 1 ml homogenates in a glass extraction vial and vortexed for 60 s 2.5 ml hexane was added to samples, and after vortexing for 60 s, tubes were centrifuged (500 g for 5 min at 4°C) to recover lipids in the upper hexane layer (aqueous phase), which was transferred to a clean tube. Aqueous samples were re-extracted as above by the addition of 2.5 ml hexane, and the upper layers were combined. Lipid extraction from the lower aqueous layer was then completed according to the Bligh and Dyer technique (34). Specifically, 3.75 ml of a 2:1 ratio of methanol: chloroform was added, followed by vortexing for 60 s. Subsequent additions of 1.25 ml chloroform and 1.25 ml water were followed with a vortexing step for 60 s, and the lower layer was recovered following centrifugation as above and combined with the upper layers from the first stage of extraction. The solvent was dried under vacuum, and lipid extract was reconstituted in 200 μl HPLC grade methanol. Lipids were separated by liquid chromatography (LC) using a gradient of 30%–100% B over 20 min (A: water: Mob B 95:5 + 0.1% acetic Acid, B: acetonitrile: methanol – 80:15 + 0.1% acetic acid) on an Eclipse Plus C18 Column (Agilent) and analyzed on a Sciex QTRAP® 6500 LC-MS/MS system. Source conditions: TEM 475 C, IS -4,500, GS1 60, GS2 60, CUR 35 (Supplemental Table S1). Chromatographic peaks were integrated using MultiQuant 3.0.2 software (Sciex) (Supplemental Fig. S1). The level of quantification (LOQ) was signal: noise of at least 5:1 (LOD 3:1) and with at least 7 points across a peak. The ratio of analyte peak areas to internal standard was taken, and lipids were quantified using a standard curve made up and run at the same time as the samples. Each oxylipin was then standardized per mg of colon tissue. For data analysis, lipids with >50% of the values below the LOQ were replaced with one-fifth of the minimum positive value of each variable and lipids where >50% of the values were below LOQ were removed from individual datasets (35).

### Fatty acid analysis

Lipids were extracted using the Bligh and Dyer methods as above, then separated using one-dimensional thin-layer chromatography (TLC) on silica gel G plates (10 × 10 cm, Merck KGaA, Darmstadt, Germany) in the solvent system hexane/diethyl ether/acetic acid (80:20:1, *v*/*v*/*v*). Plates were sprayed with a 0.05% (*w*/*l*) 8-anilino-4-naphthosulphonic acid in dry methanol and viewed under UV light to reveal lipid classes. Lipid fractions were scraped from the plates and used for fatty acid methyl ester (FAME) preparation. FAMEs were prepared by transmethylation with 2.5% H_2_SO_4_ in dry methanol ⁄ toluene (2:1, *v*/*v*) at 70°C for 2 h. A known amount of nervonic acid (C24:1n9) was added as an internal standard, so that subsequent quantification of peaks (and, consequently, lipids) could be performed. FAMEs were extracted with HPLC-grade hexane after addition of 5% aqueous NaCl. A Clarus 500 gas chromatograph with a flame ionizing detector (PerkinElmer 8,500, Norwalk, CT) and fitted with a 30 m 0.25 mm i.d. capillary column (Elite 225, PerkinElmer) was used for separation and analysis of FAs. The oven temperature was programmed as follows: 170°C for 3 min, programmed to 220°C at 4°C/min, hold for 15 min. FAMEs were identified routinely by comparing retention times of peaks with those of G411 standards (Nu-Chek Prep. Inc, Elysian, MN). PerkinElmer’s TotalChrom Navigator software was used for data acquisition of the resulting chromatographs (Supplemental Fig. S2).

### Cell culture

The murine rectal carcinoma cell line CMT-93 (ATCC) was maintained in cultured in DMEM/F-12 (Thermo Fisher Scientific) supplemented with 10% fetal bovine serum, 1% nonessential amino acids, 1% L-glutamine, 100U/ml penicillin, and 100U/ml streptomycin. Cells were confirmed as mycoplasma negative by using the LookOut® Mycoplasma PCR detection Kit (Sigma-Aldrich) and maintained for a maximum of 20 passages. Cells were seeded in 6-well plates and grown to confluency before incubating in serum-free media for 24 h before addition of 200 ng/ml 16, 16-dimethyl PGE_2_ (dmePGE_2_) ± EP2 and EP4 antagonists (PF-04418948 and ONO-AE3-208; 1 μM). After 18 h, cells were lysed in 1 × Pierce™ RIPA buffer containing 1x Halt™ Protease Inhibitor Cocktail (both Thermo Fisher Scientific) by passing through a 21-gauge needle 20–25 times on ice. The supernatant containing the protein fraction was removed by centrifugation at 9,600 g for 15 min at 4°C. Protein concentration was determined using the Pierce™ BCA Protein Assay Kit (Thermo Fisher Scientific) before Western Blot analysis.

### Western blotting

Twenty micrograms of each protein sample was resolved using a 7.5% SDS-PAGE gel before being transferred to a polyvinylidene difluoride membrane (Bio-Rad). Membranes were washed with TBS 0.2% Tween, blocked in TBS supplemented with 0.2% Tween, 3% BSA, and 0.5% gelatin for 30 min and probed with specific antibodies overnight at 4°C. After three washes of 10 min in TBS 0.2% Tween, blots were incubated with Alexa Fluor 790-conjugated Donkey anti-rabbit IgG (H + L) (Jackson ImmunoResearch) (1/10,000) for 1 h at room temperature, before imaging using the Li-Cor Odyssey cXL system (Li-Cor Biosciences). Densitometry readings were obtained using ImageJ to determine changes in protein expression. Primary antibodies used were rabbit monoclonal β-catenin (Cell Signaling Technology, #8480) and rabbit polyclonal phospho-β-catenin (Ser552) (Cell Signaling Technology, #9566), both 1/1000.

### RNA sequencing

In addition, 0.5 cm tissue samples taken from the distal colon, weighed, and added to RNAprotect tissue reagent (Qiagen) and stored overnight at −80°C. RNA was extracted using the RNeasy Mini Kit (Qiagen). RNA yield was quantified using the Qubit™ RNA BR assay kit between 185 and 289 ng/μl RNA with nanodrop spectrometry analysis revealing 260/280 nm ratio of >2 and 260/230 nm ratio of >1.9 for all samples. TapeStation Software (Agilent) revealed ribosomal integrity number of 8.3–9.5 for all samples. Samples were made up to 20 ng/μl in nuclease-free water before library construction, quality control, and Illumina next generation sequencing performed by Novogene (read length 150 bp, read depth 30 million reads) (Supplemental Table S2). Paired-end clean reads were aligned to the *Mus musculus* BALB_cJ_v1 genome available from Ensemble (EMBL-EBI) (GCA_001632525.1) using Spliced Transcripts Alignment to a Reference (STAR) software, before using the Salmon tool to quantify transcript expression. MarkDuplicates analysis revealed high duplication levels and a technical issue in RNA sequencing in two samples (Naive 4 and Hpb 4); thus, these samples were excluded from further downstream analysis. Comparison of gene expression between remaining samples was then performed using normalized counts, calculated by DESeq2 software.

### qRT-PCR

Differences in gene expression found by RNA-seq were validated by quantitative reverse transcription polymerase chain reaction (qRT-PCR). One microgram of RNA was treated with RQ1 RNase-Free DNase (Promega), before transcribed using Moloney murine leukemia virus reverse transcriptase (Invitrogen). *Alox5* and *Alox15* mRNA levels were measured by real-time PCR using an Agilent technologies Mx3000P real-time PCR machine and primers designed using Integrated DNA Technologies (NCBI Reference Sequence: NM_009660.3 and NM_009662.2). The glyceraldehyde-3-phosphate dehydrogenase (*Gapdh*) gene was used as the reference gene with primers designed using NCBI primer blast (NCBI Reference Sequence: NM_001289726.2) (primer sequences given in Supplemental Table S3). Light cycler PCR amplifications were carried out in 20 μl mixtures containing 4 μl complementary DNA, 0.5 μM primers, and 2 × PowerUp™ SYBR™ green master mix (Applied Biosystems) using the following conditions: 15 s of denaturation at 95°C, 1 min of annealing of primers at 60°C, and 1 min of elongation at 72°C, for 45 cycles. The 2ˆ-ΔΔCt method was used to calculate relative expression values between the gene of interest and the reference gene.

### Quantification and statistical analysis

Data were assessed using the GraphPad Prism 10 software (La Jolla, CA). Data were tested for normal distribution using the Shapiro-Wilk test before statistical testing. For comparison between two groups with a normal distribution, an unpaired *t* test was used, unless F test to compare variances revealed a difference in variance, at which point Welch’s correction was applied. Where three or more groups were being tested, a parametric one-way analysis of variance (ANOVA) with Tukey’s multiple comparison was applied. If data were not normally distributed, a nonparametric Mann-Whitney test or Kruskal-Wallis multiple comparison test was applied. ns on graphs denotes no statistical difference, where *P*-value ∗ = *P* < 0.05, ∗∗ = *P* < 0.01, ∗∗∗ = *P* < 0.001 and ∗∗∗∗ = *P* < 0.0001.

### Volcano plot, heatmap analysis, and pathway mapping

Volcano plots of Log2 fold change and -Log10 *P*-value were generated using GraphPad Prism 10 software, following calculation of *P*-value (see statistical analysis) and fold change between each treatment condition. Heatmaps were also generated using GraphPad Prism 10 software following calculation of fold change between treatment conditions. Pathway mapping of oxylipins was based on that generated using the WikiPathways pathway collection metabolite database: metabolites_20210109 (36) with PathVisio software (37) in addition to published reviews (38, 39).

## Results

### A high ω-6:ω-3 ratio diet increased tumor burden in a murine model of colitis-associated colorectal cancer

High dietary ratios of ω-6:ω-3 PUFAs have been linked with increased risk of colitis and colorectal cancer in both epidemiological studies and mouse models of disease (10, 11, 12, 13, 14). Although some studies have found that increasing levels of ω-3 PUFA can reduce tumor development in a preclinical model of CAC (14, 40), inconsistent results have been obtained when supplementing ω-3 or ω-6 in mouse models of colitis and spontaneous tumor formation (13, 41). Here, we address the impact of increasing the dietary ratio of ω-6:ω-3 on tumor development in a two-step model of CAC, by reducing levels of ω-3.

**Figure 1:**
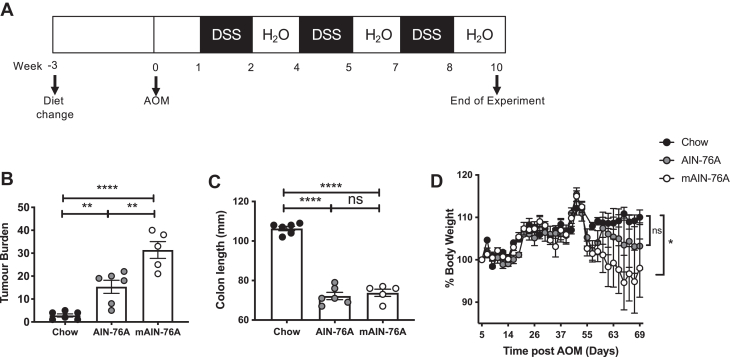
A high ω-6:ω-3 ratio diet increased colitis-associated colorectal cancer (CAC). Naive mice were fed a low ω-6 diet (chow), an AIN-76A control diet with higher ω-6 content (AIN-76A) or a modified AIN-76A diet with a higher ω-6:ω-3 ratio than AIN-76A (mAIN-76A) for three weeks before administration of 12.5 mg/kg AOM at day 0 and three fortnightly cycles of 2.5% DSS in the water (A). This diet was then continued throughout the experiment. Tumor burden (B) and colon length was quantified at day 70 following AOM. Body weight was monitored throughout the experiment with % body weight of each individual standardized to 100% at day 5 following AOM (the start of the first DSS cycle) (D). Experiments shown are one representative from two experiments with n ≥ 4 mice/group (B–D). Unpaired *t* test ∗*P* < 0.05, ∗∗*P* < 0.01, ∗∗∗*P* < 0.001, ∗∗∗∗*P* < 0.0001, error bars SEM. AIN, American Institute of Nutrition; AOM, azoxymethane; DSS, dextran sulfate sodium.

Mice were fed a chow diet lower in ω-6 PUFA (12 g/kg), a high sucrose American Institute of Nutrition (AIN)-76A rodent diet higher in ω-6 PUFA (30 g/kg, ω-6:ω-3 ratio of 43:1), or a modified AIN-76A diet with a higher ω-6:ω-3 ratio, due to lower levels of ω-3 PUFA (mAIN-76A, ω-6:ω-3 ratio of 120:1) for three weeks before induction of CAC, and continuing during the model (see Table 1 for full details of diet). The purified AIN-76A diet serves as the base diet and more appropriate control for mAIN-76A compared to the grain-based chow diet, allowing for a more precise determination of how altering ω-6:ω-3 ratio influences tumor burden. CAC was induced by administering AOM, combined with three 5-7-day cycles of DSS (Fig. 1A) and body weight was monitored throughout the experiment. At 10 weeks following administration of AOM, colon length was measured, and colon tumor burden was determined. Mice fed either AIN-76A or mAIN-76A diet, with 2.5 times the ω-6 PUFA content of chow, exhibited significantly increased tumor burden (Fig. 1B) and shortening of the colon (Fig. 1C) compared to mice fed a chow diet. Notably, tumor burden was significantly increased for mice consuming the mAIN-76A diet, with a higher ω-6:ω-3 ratio and containing less ω-3, compared to those consuming an AIN-76A diet (Fig. 1B). In agreement with increased disease severity, mice fed the mAIN-76A diet exhibited significantly increased body weight loss compared to those receiving chow (Fig. 1D).

### A high ω-6:ω-3 ratio diet increased production of LOX oxylipins derived from the ω-6 PUFA AA, while having no impact on COX-derived prostaglandins

Previous studies have found significant shifts in oxylipin production in the colon tissue of patients or animals with colorectal cancer, linking oxylipin production to the development of disease (17, 42). Increases in dietary ω-3 were proposed to reduce spontaneous colorectal cancer development by elevating basal levels of eicosapentaenoic acid (EPA) and docosahexaenoic acid (DHA) within tissue phospholipid membranes and reducing the production of proinflammatory arachidonic (AA)-derived oxylipins, including PGE_2_ (43). Since tumor development was lowest in mice fed a chow diet containing lower levels of ω-6 and highest in mice fed a mAIN-76A diet with a high ω-6:ω-3 ratio diet (Fig. 1B), we then assessed whether risk of disease was associated with changes to oxylipins and their precursors in the colon tissue after 3 weeks of feeding, and prior to disease induction.

**Figure 2:**
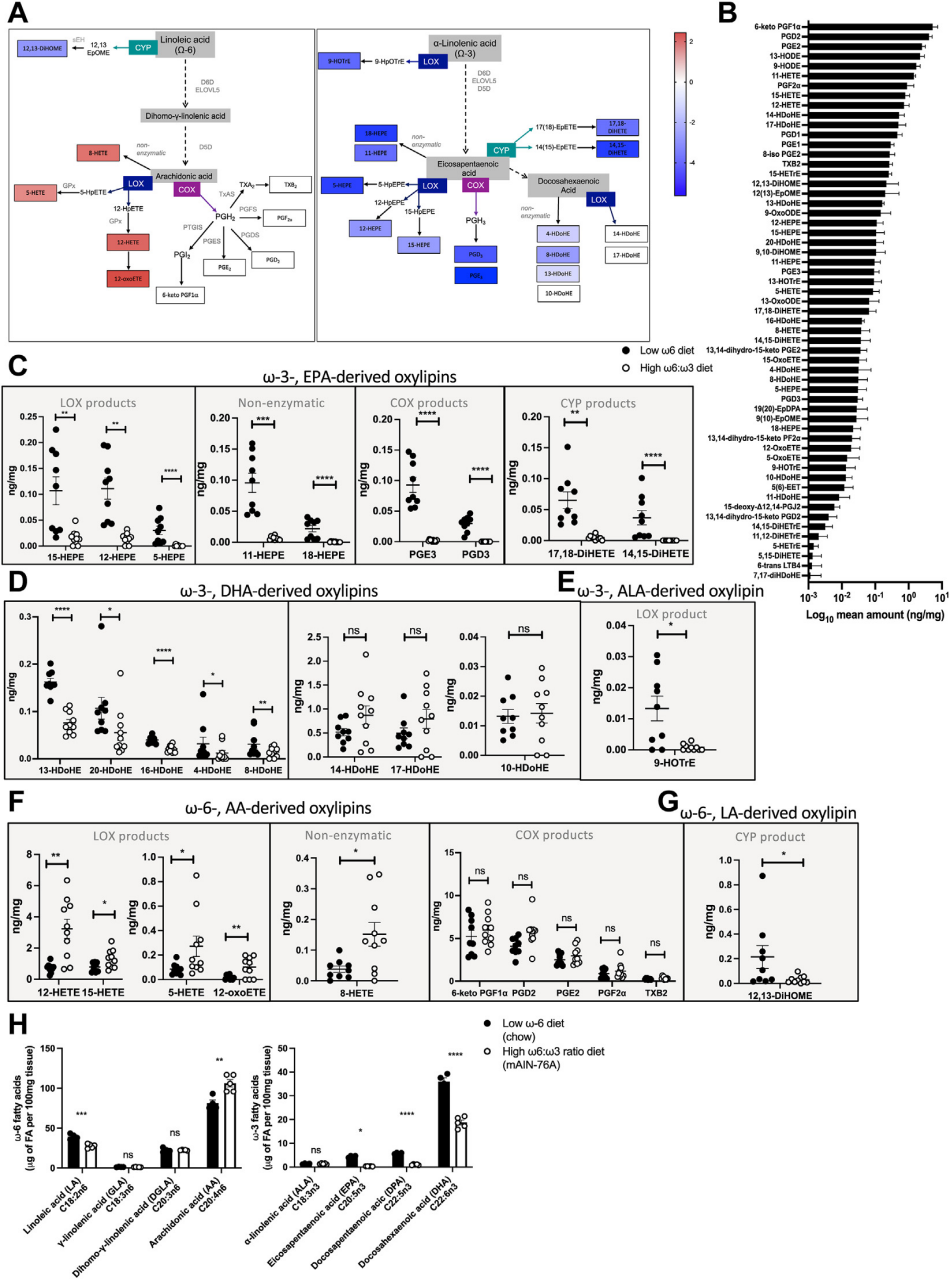
A high ω-6:ω-3 ratio diet decreased oxylipin production derived from ω-3-PUFA and increased LOX oxylipin production derived from ω-6-PUFA. Naive mice were fed a low ω-6 diet (chow), or a modified AIN-76A diet with a high ω-6:ω-3 ratio (mAIN-76A) for three weeks before lipidomic analysis of the colon. Simplified pathway representation of the Log_2_ fold change of oxylipin for mice fed a high ω-6:ω-3 ratio diet, compared to those fed a low ω-6 diet as a heatmap scaled from highest amount (*dark red*, value 2.4) to lowest amount (*dark blue*, value −5.6) (A). Mean amount of each oxylipin in the colon of mice fed a low ω-6 diet was ranked (ng/mg tissue) (B). ng per mg amount of ω-3, eicosapentaenoic acid (EPA)-derived oxylipins 15-HEPE, 12-HEPE, and 5-HEPE produced by LOX, 11-HEPE, and 18-HEPE produced nonenzymatically, PGE_3_ and PGD_3_ produced by COX and 17,18-DiHETE and 14,15-DiHETE produced by cytochrome p450 (CYP) (C). ng per mg amount of ω-3, docosahexaenoic acid (DHA)-derived oxylipins 13-HDoHE, 20-HDoHE, 16-HDoHE, 4-HDoHE, 8-HDoHE, 14-HDoHE, 17-HDoHE, and 10-HDoHE (D). ng per mg amount of ω-3, alpha-linolenic acid (ALA)-derived oxylipin 9-HOTrE (E). ng per mg amount of ω-6, arachidonic acid (AA)-derived oxylipins 12-HETE, 15-HETE, 5-HETE, 8-HETE, and 12-oxoETE produced by LOX (left panel), or 6-keto PGF1α, PGD_2_, PGE_2_, PGF2α, and TXB2 produced by COX (right panel) (F). ng per mg amount of ω-6, linoleic acid (LA)-derived oxylipin 12,13-DiHOME (G). μg per 100 mg colon tissue of the ω-6 PUFA LA, GLA, DGLA, AA, and the ω-3 fatty acids ALA, EPA, DPA, and DHA, within the total polar lipid (membrane phospholipid) fraction (H). Experiments shown are pooled data from two experiments with n ≥ 4 mice/group (A–G), or are one experiment with n ≥ 4 mice/group (H). Unpaired *t* test ∗*P* < 0.05, ∗∗*P* < 0.01, ∗∗∗*P* < 0.001, ∗∗∗∗*P* < 0.0001, error bars SEM. AIN, American Institute of Nutrition; DGLA, dihomo-γ-linolenic acid; DPA, docosapentaenoic acid; 14-HDoHE, hydroxydocosahexaenoic acid; HEPE, 5-hydroxyeicosapentaenoic; HETE, hydroxyeicosatetranoic acid; 9-HOTrE, 9-hydroxyoctadecatrienoic acid; PGE_2_, prostaglandin E_2_; 12-OxoETE, 12-oxo-eicosatetraenoic acid.

Lipidomics of the colon demonstrated that a high ω-6:ω-3 ratio diet resulted in a switch away from ω-3-derived oxylipins, to a profile dominated by oxylipins generated from the ω-6 PUFA AA by LOX (Fig. 2A). In mice fed a diet containing lower levels of ω-6, the most abundant oxylipins in the colon were 6-keto PGF1α, PGD_2_, and PGE_2_, followed by 13-hydroxyoctadecadienoic acid (HODE), 9-HODE, 11-HETE, PGF_2_α, 15-HETE, and 12-HETE (Fig. 2B). This is representative of a signature of oxylipins generated from AA by COX (6-keto PGF1α, PGD_2_, PGE_2_, PGF_2_α) and 12/15-LOX (12-, 15-HETE), or from LA by 12/15-LOX (13-HODE), and is in line with the levels of oxylipins reported in control healthy mice fed a modified AIN-93G diet (42). High levels of 14- and 17-hydroxydocosahexaenoic acid (HDoHE) in mice fed a low ω-6 chow diet are consistent with their generation from the ω-3 PUFA DHA by 12/15-LOX (Fig. 2B). 5-HETE and 5-hydroxyeicosapentaenoic (HEPE) from 5-LOX were also present at intermediate levels along with some oxylipins from cytochrome p450 (CYP), including 5(6)-epoxyeicosatrienoic acid (EET) and 12(13)-epoxyoctadecenoic acid (EpOME). Other lipids were present at lower relative amounts, particularly those with multiple oxygenations (Fig. 2B).

Following consumption of a high ω-6:ω-3 ratio mAIN-76A diet, with lower levels of ω-3, there was a significant decrease in ω-3-derived oxylipins produced by LOX, COX, and CYP (Fig. 2C–E). All HEPEs derived from EPA were significantly reduced, along with five out of eight detected HDoHEs from DHA (Fig. 2C, D left panel). Two HDoHEs generated from 12/15-LOX (14-HDoHE and 17-HDoHE) (44) and nonenzymatic oxidation product (10-HDoHE), were unaffected by the diet (Fig. 2D right panel). Levels of the ω-3, α-linolenic acid-derived oxylipin produced by LOX, 9-hydroxyoctadecatrienoic acid (9-HOTrE), were also significantly decreased by this diet (Fig. 2E). These significant decreases in ω-3-derived oxylipins were accompanied by a significant increase in several oxylipins produced by LOX from the ω-6 PUFA AA, including 5-, 12-, 15-HETE and 12-oxo-eicosatetraenoic acid (12-OxoETE) (Fig. 2F left panel). There was also a significant increase in the levels of 8-HETE, most likely produced by the non-enzymatic oxidation of AA (Fig. 2F middle panel).

Strikingly, there were no significant increases in the most abundant oxylipins produced by COX from the ω-6 PUFA AA in these mice, including 6-keto-PGF1α, PGD_2_, PGE_2_, PGF_2_α, and TXB_2_ (Fig. 2F right panel). An increase in AA-derived oxylipins was accompanied by a significant reduction in the LA-derived oxylipin produced by CYP (12,13-DiHOME) (Fig. 2G), suggesting that the generation of AA through dihomo-γ-linolenic acid (DGLA) is favored following the consumption of a high ω-6:ω-3 ratio diet. Fatty acid analysis of these samples demonstrated significantly increased levels of the ω-6 PUFA precursor AA and significantly decreased levels of the ω-3 PUFA precursors EPA, docosapentaenoic acid and DHA in the polar lipid fraction of colon samples from mice fed a high ω-6:ω-3 ratio mAIN-76A diet, when compared to mice fed a low ω-6 chow diet (Fig. 2H). The analysis demonstrated the presence of all precursors in the polar lipid fraction, representing their incorporation into membrane phospholipids. In contrast, γ-linolenic acid (GLA) and EPA were below the level of detection in the triacylglycerol fraction, representing their absence or low levels in lipid stores (Supplemental Figs. S2A, B). These data demonstrate that significant changes in oxylipin levels following dietary modification are linked to alterations in the composition of their precursors within tissue phospholipid membranes.

### Combining helminth infection with a high ω-6:ω-3 ratio diet exacerbates tumor development

**Figure 3:**
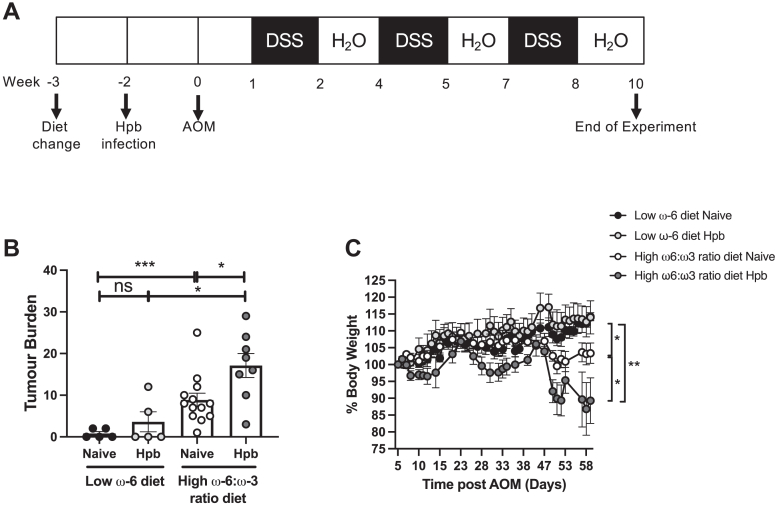
Combining helminth infection with a high ω-6:ω-3 ratio diet exacerbated tumor development. Naive mice were fed a low ω-6 diet (chow) or a modified AIN-76A diet with a high ω-6:ω-3 ratio than AIN-76A (mAIN-76A) for one week before infecting with 200 Hpb L3 larvae for 14 days (Hpb) or maintaining as naïve uninfected controls. AOM (12 mg/kg) was then administered to all groups at day 0 followed by three fortnightly cycles of 2.5% DSS in the water (A). Both diets were maintained throughout the experiment. Colon tumor burden was determined at day 59 following administration of AOM (B). Body weight was monitored throughout the course of the experiment with % body weight of each individual standardized to 100% at day 5 following AOM (the start of the first DSS cycle) (C). Experiments shown are pooled data from two separate experiments with n ≥ 4 mice/group. Unpaired *t* test ∗*P* < 0.05, ∗∗*P* < 0.01, ∗∗∗*P* < 0.001, ∗∗∗∗*P* < 0.0001. AIN, American Institute of Nutrition; AOM, azoxymethane; DSS, dextran sulfate sodium.

Adoption of high ω-6:ω-3 ratio “Western diets” is increasing in areas endemic for STH infection (30, 31). A previous study found that infection with the rodent STH *H. polygyrus bakeri* (Hpb) increased CAC in the AOM/DSS model (24); therefore, we aimed to determine how combining Hpb with differing diets might impact on tumor development. Mice fed either a low ω-6, or a high ω-6:ω-3 ratio diet were infected with Hpb after one week, then maintained on this diet throughout the experiment. CAC was induced two weeks after Hpb infection (Fig. 3A). Whereas infection of mice fed a low ω-6 chow diet resulted in a nonsignificant trend toward increased tumor burden, infection of mice fed a high ω-6:ω-3 ratio diet significantly increased tumor burden and weight loss in mice (Fig. 3B, C). Importantly, the tumor burden from combining a high ω-6:ω-3 ratio diet and Hpb infection (mean 17.1) was greater that adding the tumor burden of a high ω-6:ω-3 ratio diet (mean 8.8) to that of Hpb infection alone (mean 3.6) (Fig. 3B), suggesting an interaction, or common mechanism may be enhancing tumor development between both factors in this model.

### Combining helminth infection with a high ω-6:ω-3 ratio diet amplifies 12/15-LOX oxylipin production and increases expression of Alox15 and Alox5

**Figure 4:**
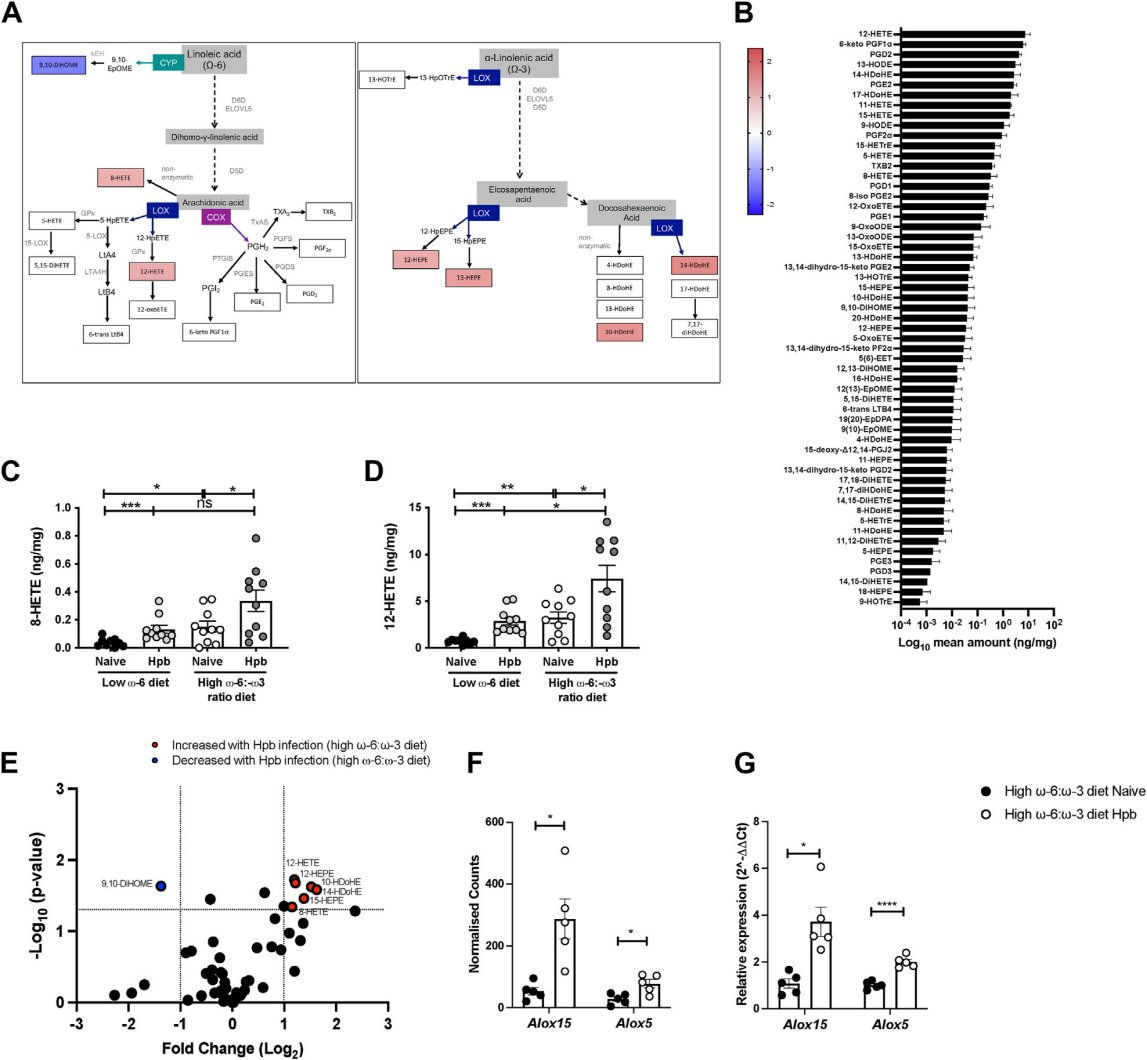
Combining helminth infection with a high ω-6:ω-3 ratio diet amplified 12/15-LOX oxylipin production and increased expression of *Alox15* and *Alox*5. Naive mice were fed a low ω-6 diet (chow) or a modified AIN-76A diet with a high ω-6:ω-3 ratio than AIN-76A (mAIN-76A) for one week, then infected with 200 Hpb L3 larvae for 14 days (Hpb), or maintained as naïve uninfected controls, before lipidomic or RNA-seq analysis of the colon. Simplified pathway representation of the Log_2_ fold change of oxylipin for helminth infected mice fed a high ω-6:ω-3 ratio diet, compared to naïve mice fed a low ω-6 diet as a heatmap scaled from highest amount (*dark red*, value 2.4) to lowest amount (*dark blue*, value −2.3) (A) Mean amount of each oxylipin in the colon of helminth infected mice fed a high ω-6:ω-3 ratio diet was ranked (ng/mg tissue) (B). ng per mg amount of the oxylipins 8-HETE (C) and 12-HETE (D). Volcano plot of all colon oxylipins, highlighting those significantly increased (*red* symbols) or decreased (*blue* symbols) in helminth infected mice fed a high ω-6:ω-3 ratio diet, compared to naïve uninfected mice fed a high ω-6:ω-3 ratio diet (E). *Dotted line* represents -Log_10_*P*-value <0.05 (y-axis) and Log_2_ Fold Change >2 (x-axis). Expression levels of *Alox15* and *Alox5* (F) and relative expression of *Alox15* and *Alox5* (G), in the colon of naïve and helminth infected mice fed a high ω-6:ω-3 ratio diet, taken at day 14 post infection. Experiments shown are pooled data from two separate experiments with n ≥ 4 mice/group (A–E), or one experiment with n = 5 mice/group (F and G). Unpaired *t* test ∗*P* < 0.05, ∗∗*P* < 0.01, ∗∗∗*P* < 0.001, ∗∗∗∗*P* < 0.0001, error bars SEM. AIN, American Institute of Nutrition; HETE, hydroxyeicosatetranoic acid.

Infection with a rodent helminth, or exposure to Hpb products, increased production of proinflammatory ω-6, AA-derived oxylipins in vivo, or in vitro models using murine and human macrophages (27, 28). Hpb infection and exposure to Hpb products also increased the expression of oxylipin-generating enzymes in vivo and in *vitro* (28, 45, 46). After finding that AA-derived oxylipins produced by LOX correlate with dietary-dependent increases in tumor development (Figs. 2A, 1B), we were motivated to determine whether similar changes occurred during helminth exacerbation of disease. Corresponding to what was seen with a high ω-6:ω-3 ratio diet, Hpb infection of mice fed a high ω-6:ω-3 ratio diet resulted in significantly increased production of AA-derived oxylipins produced by 12/15-LOX, with 12-HETE becoming the most abundant oxylipin found in the colon (Fig. 4A, B). The combination of Hpb infection and a high ω-6:ω-3 ratio diet had a synergistic effect on 8-HETE and 12-HETE levels, both derived from AA by either nonenzymatic oxidation or 12/15-LOX, respectively (Fig. 4C, D). Unlike mice fed a high ω-6:ω-3 ratio diet alone, Hpb infection of mice fed a high ω-6:ω-3 ratio did not result in a shift away from ω-3-derived oxylipins and only production of 9,10-DiHOME, a CYP product of LA, was significantly reduced (Fig. 4E). Instead, infection significantly increased production of several ω-3-derived oxylipins, including the 12/15-LOX products 12-HEPE, 15-HEPE, and 14-HDoHE, and the nonenzymatic oxidation product 10-HDoHE (Fig. 4A–E). Of note, 12/15-LOX oxylipin levels were also significantly increased in the colon of Hpb-infected mice fed a low ω-6 diet (Fig. 4D, Supplemental Fig. S3D, E). As these mice were not prone to a significantly increased tumor development (Fig. 3B), our data suggest that tumor burden was not solely linked to increased basal levels of the 12/15-LOX-derived oxylipins, following Hpb infection.

Because infection with the helminth *Taenia crassiceps* was previously shown to increase *Alox15* expression in peritoneal macrophages in response to interleukin (IL)-4 signaling (47), we hypothesized that Hpb may similarly promote 12/15-LOX oxylipin production through this pathway. RNA-seq and qRT-PCR validation of colon tissue demonstrated that expression of *Alox15* and *Alox5* was significantly increased in Hpb-infected mice fed a high ω-6:ω-3 ratio diet when compared to mice fed a high ω-6:ω-3 ratio diet alone (Fig. 4F, G). However, expression of *Alox12*, *Alox12e*, *Alox8, Alox12b*, *Il13rα1, Il4rα, Il2rg*, *and Il13* were not altered following Hpb infection (Supplemental Fig. S3A–C).

Consistent with what was seen in mice fed a high ω-6:ω-3 ratio diet alone, the production of the most abundant AA-derived prostaglandins PGD_2_, 6-keto-PGF1α, PGE_2_, and PGF2α, was not significantly altered by Hpb infection of mice fed a low ω-6, or high ω-6:ω-3 ratio diet (Supplemental Fig. S3E–I). Expression of *Ptgs1*, *Ptgs2*, and *Ptges1* were similarly unaffected in the colon of Hpb-infected mice fed a high ω-6:ω-3 ratio diet (Supplemental Fig. S3J). As binding of PGE_2_ to the PGE_2_ receptor 2 (EP2), or EP4, can promote colorectal cancer (48, 49), the contribution of prostaglandin signaling to helminth-exacerbation of tumor formation was next assessed.

### Aspirin administration significantly reduces Hpb-dependent exacerbation of CAC but has no impact on 12/15-LOX-derived oxylipin production

**Figure 5:**
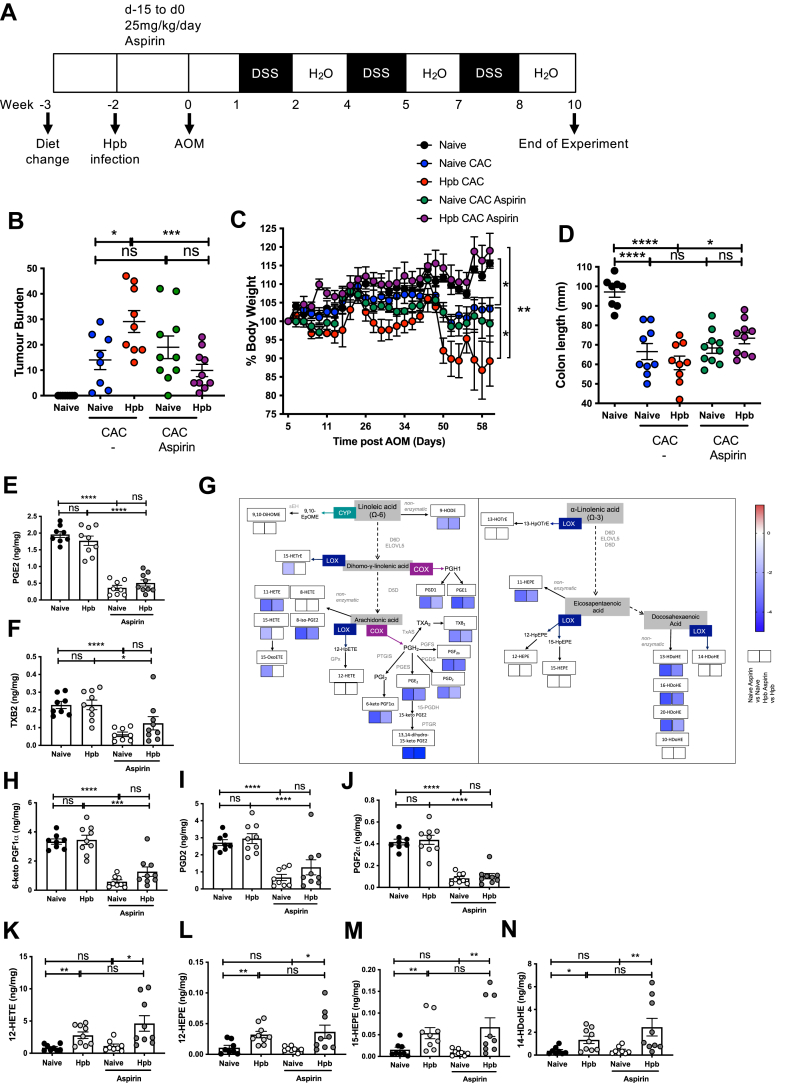
Administration of aspirin during helminth infection significantly reduced tumor burden and COX-dependent oxylipin production derived from AA. All groups of mice were fed a high ω-6:ω-3 ratio diet throughout the experiment. One group was infected with 200 Hpb L3 larvae for 14 days (Hpb) (*red* symbols) and one group was maintained as uninfected (naive) controls (*black* symbols). 25 mg/kg/day aspirin was delivered in the water from day −15 to day 0 of Hpb infection (*purple* symbols), or the equivalent time to uninfected (naive) mice (*green* symbols). Hpb-infected (*red* symbols) and uninfected (naive) mice (*blue* symbols) were maintained on normal drinking water. At day 0, aspirin-treated groups were placed onto water and CAC was initiated by administering AOM at day 0, followed by three fortnightly cycles of DSS in the water (A). Tumor burden was quantified in the colon at day 59 following administration of AOM (B). Body weight was monitored throughout the experiment with % body weight of each individual standardized to 100% at day 5 following AOM (the start of the first DSS cycle) (C). Colon length was quantified in the colon at day 59 following administration of AOM (D). ng per mg amount of the oxylipins PGE_2_ (E) and TXB_2_ (F) were quantified in the colon at day 0. Simplified pathway representation of the Log_2_ fold change of oxylipins for uninfected (naive) mice treated with aspirin, compared to uninfected (naive) naïve maintained on water, or Hpb-infected mice treated with aspirin, compared to Hpb-infected mice maintained on water as a heatmap scaled from highest amount (dark *red*, value 1.4) to lowest amount (dark *blue*, value −5) (G). ng per mg amount of the oxylipins 6-keto PGF1α (H), PGD_2_ (I), PGF_2_α (J), 12-HETE (K), 12-HEPE (L), 15-HEPE (M), and 14-HDoHE (N) in the colon at day 0. Experiments shown are pooled data from two experiments with n ≥ 4 mice/group. Unpaired *t* test ∗*P* < 0.05, ∗∗*P* < 0.01, ∗∗∗*P* < 0.001, ∗∗∗∗*P* < 0.0001, error bars SEM. AOM, azoxymethane; CAC, colitis-associated colorectal cancer; DSS, dextran sulfate sodium; HEPE, 5-hydroxyeicosapentaenoic; HETE, hydroxyeicosatetranoic acid; 14-HDoHE, hydroxydocosahexaenoic acid; PGE_2_, prostaglandin E_2_.

Aspirin is thought to reduce the risk of colorectal cancer by inhibiting COX-dependent prostaglandin production and subsequent prostaglandin receptor signaling (48). To determine the contribution of prostaglandin signaling to helminth exacerbation of CAC, aspirin was administered at 25 mg/kg/day in the drinking water starting one day before Hpb infection and continuing throughout the 14-day infection period. Mice were then restored to normal water and CAC was initiated by injecting AOM (Fig. 5A). Uninfected (naive) mice fed a high ω-6:ω-3 ratio diet were also given aspirin for the equivalent period before injecting AOM, to determine any effect of treatment on CAC initiation. Aspirin treatment of Hpb-infected mice significantly reduced their heightened tumor burden, to the levels observed in uninfected mice fed a high ω-6:ω-3 ratio diet with CAC (Fig. 5B, Supplemental Fig. S4A, B). Weight loss and colon shortening were also significantly reduced following aspirin treatment of Hpb-infected mice, when compared to Hpb-infected mice receiving water (Fig. 5C, D). Continuous administration of 25 mg/kg/day aspirin during the AOM/DSS limited tumor development in mice, whereas administration from 4 days before AOM to 4 days after AOM had no significant impact on tumor formation (15). In line with these findings, aspirin delivery for 2-weeks before AOM administration did not alter tumor formation, weight loss, or colon shortening in uninfected mice, suggesting that COX-dependent oxylipins were not involved in CAC development driven by a high ω-6:ω-3 ratio diet (Fig. 5B–D, Supplemental Fig. S4A, B).

Lipidomics analysis of the colons from mice given aspirin for two weeks during helminth infection, or the equivalent period in uninfected controls, showed that aspirin significantly reduced levels of PGE_2_ and TXB_2_ in mice fed a high ω-6:ω-3 ratio diet, prior to disease onset (Fig. 5E, F). PGE_2_ levels were similarly reduced in Hpb-infected mice following aspirin treatment, although TXB_2_ was less affected (Fig. 5E, F). Pathway representation showed that aspirin suppressed levels of all other COX-derived oxylipins in both uninfected and Hpb-infected mice (Fig. 5G), including the more abundant ω-6-derived oxylipins 6-keto-PGF1α, PGD_2_, and PGF_2_α (Fig. 5H–J), as well as the lower abundance oxylipins PGD1, PGE1, and 13,14-dihydro-15-keto PGE_2_ (Supplemental Fig. S4C–E). Eleven-HETE (a nonenzymatic product from AA) and 9-HODE (a nonenzymatic product from LA) were also significantly reduced by aspirin treatment as well as the low abundance ω-3-derived oxylipins, 11-HEPE, 13-, 16-, and 20-HDoHE (Supplemental Fig. S4F–K), which may be indicative of aspirin’s ability to inhibit nonenzymatic oxidative stress (50). However, aspirin did not alter the increased production of 12/15-LOX products 12-HETE, 12-HEPE, 15-HEPE, or 14-HDoDE found in Hpb-infected mice (Figs. 5K–N, 4A). Aspirin significantly reduced the production of the ω-6-derived oxylipins 15-HETE, 15-oxoETE, and 15-HETrE in naive mice, but not in Hpb-infected mice (Supplemental Fig. S4L–N). 15-HETE is formed as the S-enantiomer by 12/15-LOX, or as a mixture of the 15(S)- and 15(R)-forms, by COX-1. Aspirin blocks COX-1-dependent production of 15(S)-HETE by platelets (51) and COX-2-dependent production of 15(R)-HETE (52). Therefore, our results suggest that 15-HETE is likely generated by COX in uninfected mice, whereas it is more likely to be generated by 12/15-LOX (53), following Hpb infection. In summary, aspirin inhibited COX-dependent oxylipin biosynthesis, while having no impact on 12/15-LOX products.

### Enhanced prostaglandin receptor signaling exacerbated CAC and was increased following helminth infection of mice fed a high ω-6:ω-3 ratio diet

**Figure 6:**
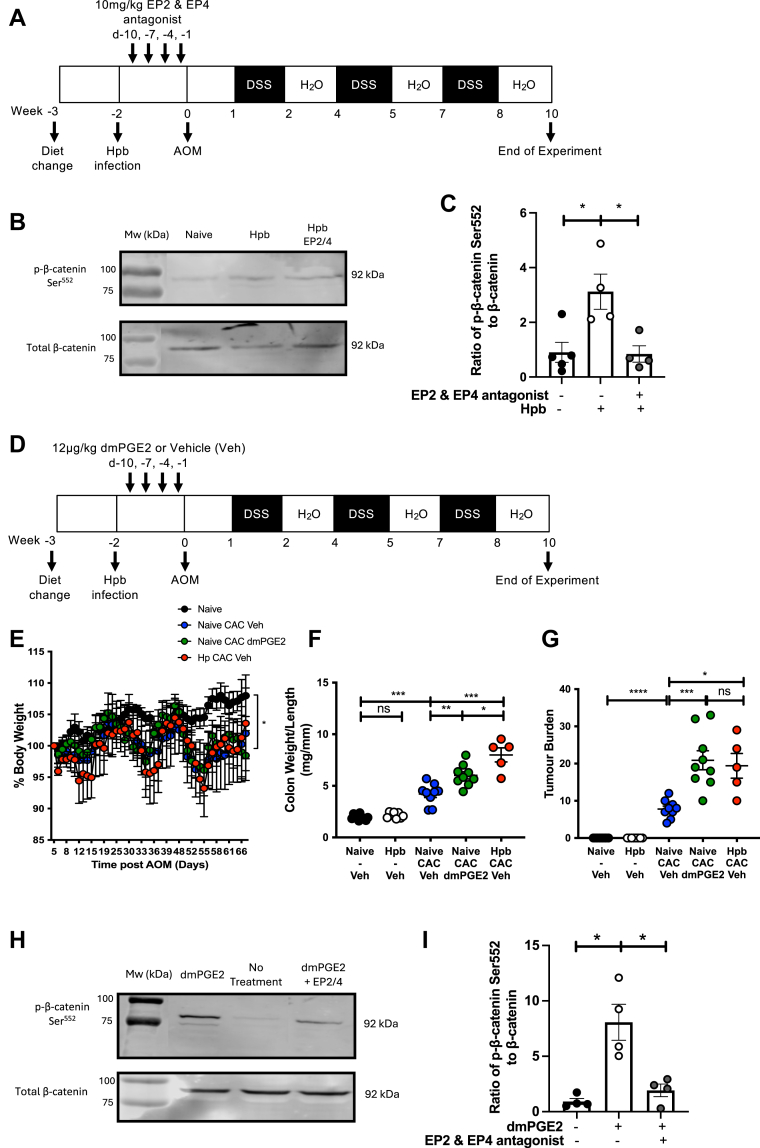
Enhanced prostaglandin receptor signaling increased CAC, to the same extent as helminth infection. All groups of mice were fed a high ω-6:ω-3 ratio diet throughout the experiment. To determine whether helminth infection activated EP signaling, Hpb-infected mice were given 10 mg/kg of the EP2 and EP4 antagonists PF-04418948 and ONO-AE3-208 i.p. at day −10, −7, −4, and −1 before AOM administration (Hpb EP2/4). Uninfected (Naive) and Hpb-infected (Hpb) were given 1:1 DMSO:PBS i.p. as a vehicle control. At day 0, CAC was initiated by administering AOM at day 0, followed by three fortnightly cycles of DSS in the water (A). Example Western blot of p-β-catenin Ser552 and total β-catenin from the colon of mice for each treatment condition day 64 following administration of AOM (B). Graphical summary of the ratio of p-β-catenin Ser552 levels to total β-catenin levels for all treatment conditions (C). To determine the influence of EP activation on tumor burden, one group of mice was infected with 200 Hpb L3 larvae for 14 days (Hpb) (*red* symbols) and one group was maintained as uninfected (naive) controls (*black* symbols). Uninfected (naive) mice were given 12 μg/kg of the EP agonist 16,16-dimethyl PGE_2_ (dmPGE2) i.p. at day −10, −7, −4, and −1 before AOM administration (*green* symbols). Hpb-infected (*red* symbols) or uninfected (naive) mice (*blue* symbols) were given 1:1 DMSO:PBS i.p. as a vehicle control (Veh). At day 0, CAC was initiated by administering AOM at day 0, followed by three fortnightly cycles of DSS in the water (D). Body weight was monitored throughout the experiment with % body weight of each individual standardized to 100% at day 5 following AOM (the start of the first DSS cycle) (E). Colon weight to length ratio (mg/mm) (F) and tumor burden (G) were determined at day 61 following administration of AOM in Hpb-infected or uninfected (naive) mice receiving dmPGE2 or a vehicle control (Veh). The impact of EP2/EP4 antagonists (PF-04418948, ONO-AE3-208; 1 μM) on dmPGE_2_-induced increases in phosphorylation of β-catenin at Ser552 was assessed in a CMT-93 cell line. Example Western blot of p-β-catenin Ser552 and total β-catenin from CMT-93 cells treated with dmPGE2 (200 ng/ml) ± EP2/4 inhibitors (1 μM) (H). Graphical summary of the ratio of p-β-catenin Ser552 levels to total β-catenin levels for all treatment conditions (I). Experiments shown are pooled data from two separate experiments with n ≥ 4 mice/group (E–G), are one experiment with n = 5 mice/group (B and C), or are pooled data from four separate experiments with n = 1/group (I). Unpaired *t* test ∗*P* < 0.05, ∗∗*P* < 0.01, ∗∗∗*P* < 0.001, ∗∗∗∗*P* < 0.0001, error bars SEM. AOM, azoxymethane; CAC, colitis-associated colorectal cancer; DSS, dextran sulfate sodium; PGE2, prostaglandin E_2_.

The expression of nuclear p-β-catenin Ser^552^ has been linked to disease progression in the CAC AOM/DSS mouse model (54). COX-dependent oxylipin PGE_2_ is reported to promote tumor development in *Apc*^*Min/+*^ mice through EP2/4-dependent phosphorylation of β-catenin at Ser552 (p-β-catenin Ser^552^) (55). As oral administration of the EP2 antagonist and oral delivery of the EP4 antagonist ONO-AE3-208 significantly reduced tumor burden and DSS-induced colitis, respectively, when given during disease development (48, 56), we hypothesized that Hpb exacerbation of CAC in mice fed a high ω-6:ω-3 ratio diet may be due to increased EP2/4-dependent signaling. The ratio of p-β-catenin Ser^552^ to β-catenin was quantified at day 64 following administration of AOM in Hpb-infected mice given a vehicle control (Veh) to Hpb-infected mice administered prostaglandin E_2_ receptor 2 (EP2) and EP4 antagonists (PF-04418948, ONO-AE3-208; 10 mg/kg) i.p. during Hpb infection (Fig. 6A). Supporting the hypothesis, the ratio of p-β-catenin Ser^552^ to β-catenin was significantly increased in helminth-infected mice fed a high ω-6:ω-3 diet at day 64 following administration of AOM, when compared to naive controls (Fig. 6B, C, Supplemental Fig. S5A, B). Significantly, administration of EP2/4 antagonists to Hpb-infected mice prevented the helminth-driven phosphorylation of β-catenin at Ser552 (Fig. 6B, C, Supplemental Fig. S5A, B). To confirm our hypothesis, the PGE_2_ analogue 16, 16-dimethyl PGE_2_ (diMe-PGE_2_) was administered to uninfected mice on a high ω-6:ω-3 diet, at days −10, −7, −4, and −1, followed by AOM administration (Fig. 6D). This is a competitive inhibitor of 15-hydroxy prostaglandin dehydrogenase (15-PGDH), which slows metabolism of PGE_2_, and acts as an EP2, 3 and 4 agonist (57, 58). In the CAC model, diMe-PGE_2_ significantly increased colon weight-to-length ratio and tumor burden of uninfected mice, reaching the level experienced by Hpb-infected mice (Veh) (Fig. 6E–G). This indicates that PGE_2_ signaling can increase tumor burden in a manner similar to the impact of Hpb infection in the CAC model (Fig. 6G). Furthermore, these data provide further evidence that the impact of Hpb on tumor burden is mediated through increased PGE_2_ receptor signaling, akin to diMe-PGE_2_ administration. Since increased phosphorylation of β-catenin at Ser552 was observed following exposure of a human colorectal cancer cell line to PGE_2_ (55), we determined whether dmPGE_2_ mediated this effect through EP2/4-dependent signaling. Using a murine colorectal cancer cell line, the ratio of p-β-catenin Ser^552^ to β-catenin was increased following exposure to dmPGE_2_. This effect was effectively inhibited by EP2/4 antagonists (Fig. 6H, I, Supplemental Fig. S6A, B). These results provide further support that dmPGE2 and Hpb infection promote tumorigenesis through EP2/4-signaling.

## Discussion

Epidemiological evidence indicates that helminths or a high ω-6:ω-3 PUFA diet are independently associated with an increased risk of colorectal cancer in humans (10, 22, 25, 59, 60), however up until now, it was not known whether they could act in concert to further drive risk. Furthermore, how these two independent factors might mechanistically interact to influence the risk of CAC was not known. This is important since populations where helminth infections are most common are increasingly switching to high ω-6 diets (30, 31). Here, using a mouse model of CAC, we combine high ω-6:ω-3 diet with a rodent helminth infection and show a significantly increased tumor burden, which is mechanistically linked to helminth driven-PGE_2_ signaling via EP2/4 receptors. Although 12/15-LOX-derived oxylipin production from AA was elevated by both conditions, it was clear that basal levels of these oxylipins did not correlate with tumor burden. Because exacerbation of disease only occurs when both factors were combined, our study proposes a direct interaction between two independent risk factors for CAC, one which is modifiable by diet and the other which is targetable through COX inhibition of oxylipin production.

First, we explored whether high ω-6:ω-3 PUFA ratios could impact the development of colorectal cancer using a preclinical CAC model, finding that reducing ω-3 content is linked to increased tumor burden. This is consistent with previous studies that have modified ω-6:ω-3 ratios through supplementation of the diet with ω-3, or the use of *fat-1* transgenic mice, which convert dietary ω-6 to ω-3 PUFAs (13, 14, 40, 43, 61, 62). These findings are significant because a shift toward a high ω-6:ω-3 ratio diet ranging between 20:1 and 50:1 in the West has been linked to the development of chronic inflammatory diseases, including colorectal cancer (9, 63, 64, 65, 66). In low-HDI regions, the estimated lower ω-6:ω-3 ratio of 1:1-4:1 of traditional diets in rural areas has reflected lower colorectal cancer incidence (67). However, dietary patterns in these low-HDI regions are shifting toward to a Western ω-6:ω-3 ratio, coinciding with an estimated increase in colorectal cancer cases (1, 30, 31). Our data highlight the importance of maintaining a balanced ω-6:ω-3 PUFA ratio in both regions, to reduce the risk of cancer.

Our study further examined mechanisms driving tumor formation by measuring diet-driven levels of oxylipins in the colon, prior to tumor development. We found that a high ω-6:ω-3 ratio diet resulted in a switch away from ω-3-derived oxylipins, to a profile dominated by oxylipins generated from the ω-6 PUFA AA by LOX, but not COX. As increases in dietary ω-3 PUFAs have previously been linked with a reduction in cancer development through lowering of AA levels within tissue phospholipid membranes and a decrease in the production of proinflammatory AA-derived prostaglandins (PG) (43), our results suggest that increased tumor burden in mice fed a high ω-6:ω-3 ratio diet may be driven by increased production of proinflammatory AA-derived oxylipins produced by LOX, due to reduced availability of ω-3-derived EPA and DHA substrate. Indeed, fatty acid analysis confirmed significantly increased AA and significantly reduced EPA and DHA in the colon of mice fed a high ω-6:ω-3 ratio diet. The lack of difference seen for AA-derived oxylipins produced by COX and their high abundance in the colon, may imply that COX is already saturated with AA, before switching to a high ω-6:ω-3 ratio diet. Diet-driven alterations to these pathways have been implicated in promoting tumor development, although given the nuances of different model types and sampling locations for oxylipins, it has not yet been possible to draw definitive conclusions linking changes in basal levels of oxylipins in the colon to disease progression (40, 43, 61). Although it remains to be determined how diet-induced alterations in basal levels of oxylipin production in the colon from a high ω-6:ω-3 diet are directly linked to tumor development, these findings underscore the need for dietary modifications to increase ω-3 intake and manage ω-6 levels, thereby reducing CRC development.

We then explored how helminth infection could further influence colorectal cancer in animals fed a low ω-6, or a high ω-6:ω-3 ratio diet. In a previous study, infection with *H. polygyrus bakeri* significantly increased tumor burden in the AOM/DSS mouse model of CAC (24). However, in our study, helminth infection of mice fed a low ω-6 diet did not result in a significant increase in tumor burden, nor did we observe high tumor burden in uninfected mice. This discrepancy may be due to the use of a mouse strain with reduced susceptibility to CAC or colitis (68, 69, 70), or to differences in the microbiota of mice maintained on a grain-based low ω-6 chow diet (71, 72). Notably, an increased tumor burden was observed in uninfected mice following the introduction of a modified AIN-76A diet, with a further increase upon helminth infection. These findings suggest that diet-dependent alterations in the microbiota may underlie the regulation of disease seen in our model. When helminth infection was combined with a high ω-6:ω-3 ratio diet, we observed further increases in the basal levels of 12/15-LOX oxylipins derived from AA. Whereas a shift in 12/15-LOX oxylipin production in mice fed a high ω-6:ω-3 ratio diet is likely due to the availability of substrate for the metabolic enzyme and saturation of enzymatic activity, these increases were likely mediated by increased *Alox15* expression following helminth infection. However, because 12/15-LOX oxylipins were significantly increased in the colon of helminth-infected mice fed a low ω-6 diet, which were not prone to increased tumor development, our data suggest that tumor burden was not solely linked to increased expression of *Alox15*, or the basal level of the 12/15-LOX-derived oxylipins following Hpb infection. Instead, helminth exacerbation of disease is more likely due to a coordinated response between proinflammatory signaling pathways activated by the AA-derived oxylipins favored by a high ω-6:ω-3 ratio diet (eg 12-HETE and 8-HETE), and those promoted by helminth infection.

Like a high ω-6:ω-3 ratio diet, helminth infection of mice on a high ω-6:ω-3 ratio diet had no effect on COX-derived prostaglandin production in the colon, at least not at the time points measured. Direct exposure to Hpb larval antigens triggered rapid production of PGE_2_ within 24 h (28), although all prostaglandins are reported to have a short half-life, rapidly being oxidized and inactivated by 15-hydroxyprostaglandin dehydrogenase (15-PGDH) (73). By day 14 following infection with Hpb, the peak of prostaglandin production may have subsided and mice would be recovering from acute inflammation dominated by the production of LOX-derived oxylipins, as reported during the recovery phase of DSS treatment (74). Therefore, an extensive time course would be needed to determine how Hpb infection in the small intestine influences prostaglandin production in the colon. Despite this, inhibition of COX-derived prostaglandin production with aspirin inhibited helminth-exacerbation of CAC, unlike the increased tumor burden seen in mice fed a high ω-6:ω-3 ratio diet alone. Because the impact of aspirin on prostaglandin levels was broadly similar regardless of Hpb infection, this finding supports the hypothesis that helminth exacerbation of tumor burden is not solely dependent on oxylipin production but may result due to coordination between downstream receptor signaling mediated by COX-derived products following Hpb infection, and proinflammatory signaling pathways activated by AA-derived oxylipins favored by a high ω-6:ω-3 ratio diet. Aspirin treatment had no impact on increased 12/15-LOX oxylipins in helminth-infected mice, supporting the hypothesis that exacerbation of tumor burden was not dependent on Hpb-increases in *Alox15* expression and production of 12/15-LOX oxylipins alone. Although our study did not identify the cellular sources of specific oxylipins, previous research has highlighted the role of monocytes in producing COX-derived, but not LOX-derived oxylipins, following exposure to helminth antigens (28, 29). The expansion of 12/15-LOX expressing CD11b^+^Gr-1^+^ myeloid suppressor cells following helminth infection suggests that distinct cell types are responsible for LOX- and COX-derived oxylipin production in this context (47); however, further research is needed to confirm this.

Helminth infection was associated with increased activation of PGE_2_ receptor EP2/EP4 signaling and administration of an EP receptor agonist increased tumor burden in naïve mice fed a high ω-6:ω-3 diet. These findings clearly implicate helminth activation of PGE_2_ signaling in tumor formation via the EP2 and EP4 receptors. Signaling via EP2 and EP4 promoted tumor formation or colitis (48, 56), and we add to this by showing that helminth activation of EP2/4 is associated with increased tumor development. However, further research would be required to confirm whether helminth infection increases tumorburden through this pathway. A previous study has shown that exposure to the antigens of *T. solium* cysts can activate EP2 and EP4, thereby setting the precedent for helminth-driven increase in PGE_2_ signaling (29). Activation of EP2/4 following exposure to this antigen is ascribed to a helminth glutamate dehydrogenase, also present in the larval antigens of Hpb (28, 29). Recent evidence shows that helminth glutamate dehydrogenase-dependent alterations in oxylipin production by macrophages are mediated through activation of p300 histone acetyltransferase and epigenetic regulation of immune regulatory genes (75). Although oxylipin signaling in rodents may differ in some respects from that in patients, many of our major findings are compatible with human studies. This includes the elevation 12/15- and 5-LOX derived oxylipins in tissue of colorectal cancer, and the lack of impact of aspirin supplementation (17) (Figs. 2A, 4A and 5G), the increased phosphorylation of β-catenin on Ser-552 in patients with colorectal cancer (reviewed in (76)) and the activation of EP2/EP4 signaling following human helminth infection (reviewed in (77)) (Fig. 6C). Although an epidemiological study would be required to determine whether a similar pathway occurs following Hpb infection in humans, these findings suggest that targeting prostaglandin signaling may help prevent the rising incidence of CAC in helminth-endemic areas adopting a “Western diet”.

A key message of this study is that exacerbation of tumor burden is only seen when we combine Hpb infection with a high ω-6:ω-3 ratio diet. This suggests that two interlinked signals, one resulting from dietary-related increases in AA-derived LOX oxylipins and the other resulting from increased EP2/4 signaling due to Hpb infection, likely coordinate to result in exacerbation of disease. Further work is now needed to deconvolute the precise nature of this interaction. Given the challenges of deworming strategies and the delivery of aspirin to rural areas within low-HDI regions, it may be more effective to promote dietary modification as a strategy to prevent CAC in helminth endemic areas. Our data provide support that this strategy may mitigate the exacerbation of CAC due to the interaction between dietary changes and helminth infection in these regions.

## Data availability

Data sources and handling of the publicly available datasets used in this study are described in the Materials and Methods. Example LC/MS/MS chromatograms, multiple reaction monitoring transitions, GC-FIT chromatographs, qRT-PCR primer details and raw Western blots are contained within the supplemental information of the article. The RNA-seq data reported in this study are available at the Gene Expression Omnibus under the accession code GSE298815. Further information is available from the corresponding author upon request.

## Supplemental data

This article contains supplemental data.

## Conflict of interest

The authors declare that they have no conflicts of interest with the contents of this article.
