# Supplementary material for: Helminth-induced prostaglandin signaling and dietary shifts in PUFA metabolism promote colitis-associated cancer
Source: J Lipid Res. 2025 Jun 7;66(7):100837. doi: 10.1016/j.jlr.2025.100837 (PMC12272432; doi:10.1016/j.jlr.2025.100837)
Supplement: Supplemental Information [file mmc1.pdf]

SUPPLEMENTAL INFORMATION:

Helminth-induced prostaglandin signalling and dietary shifts in PUFA metabolism promote colitis-associated cancer.

Katherine A. Smith[1,*], smithk28@cardiff.ac.uk, Ella K. Reed[1], Irina Guschina[1], Victoria J. Tyrrell[5], Claire Butters[2], Matthew G. Darby[2], Brunette Katsandegwaza[3], Alisha Chetty[2], William G.C. Horsnell[4], Valerie B. O'Donnell[5], Awen Gallimore[5]

[1]Cardiff University, School of Biosciences, Cardiff, UK

[2]University of Cape Town, Institute of Infectious Disease and Molecular Medicine, Cape Town, South Africa

[3]University of Liege, Department of Infectious and Parasite Diseases, Liege, Belgium

[4]University of Exeter, Medical Research Council Centre for Medical Mycology, Exeter, UK

[5]Cardiff University, School of Medicine, Cardiff, UK

*For correspondence: Katherine A Smith

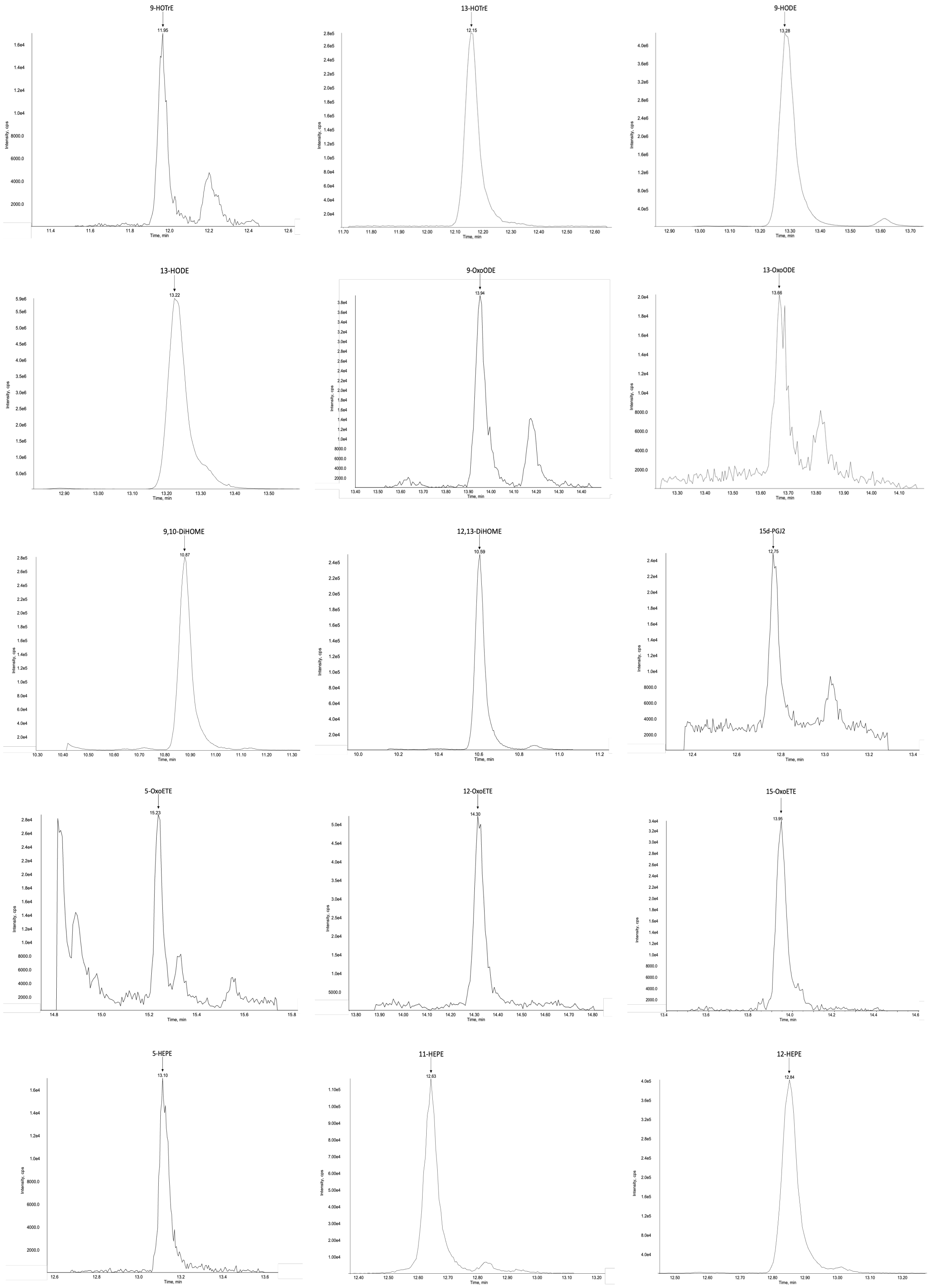
9-HOTrE
11.95
13-HOTrE
12.15
9-HODE
13.28
13-HODE
13.22
9-OxoODE
13.94
13-OxoODE
13.66
9,10-DiHOME
10.87
12,13-DiHOME
10.59
15d-PGJ2
12.75
5-OxoETE
15.23
12-OxoETE
14.30
15-OxoETE
13.95
5-HEPE
13.10
11-HEPE
12.63
12-HEPE
12.84
Intensity, cps
Time, min

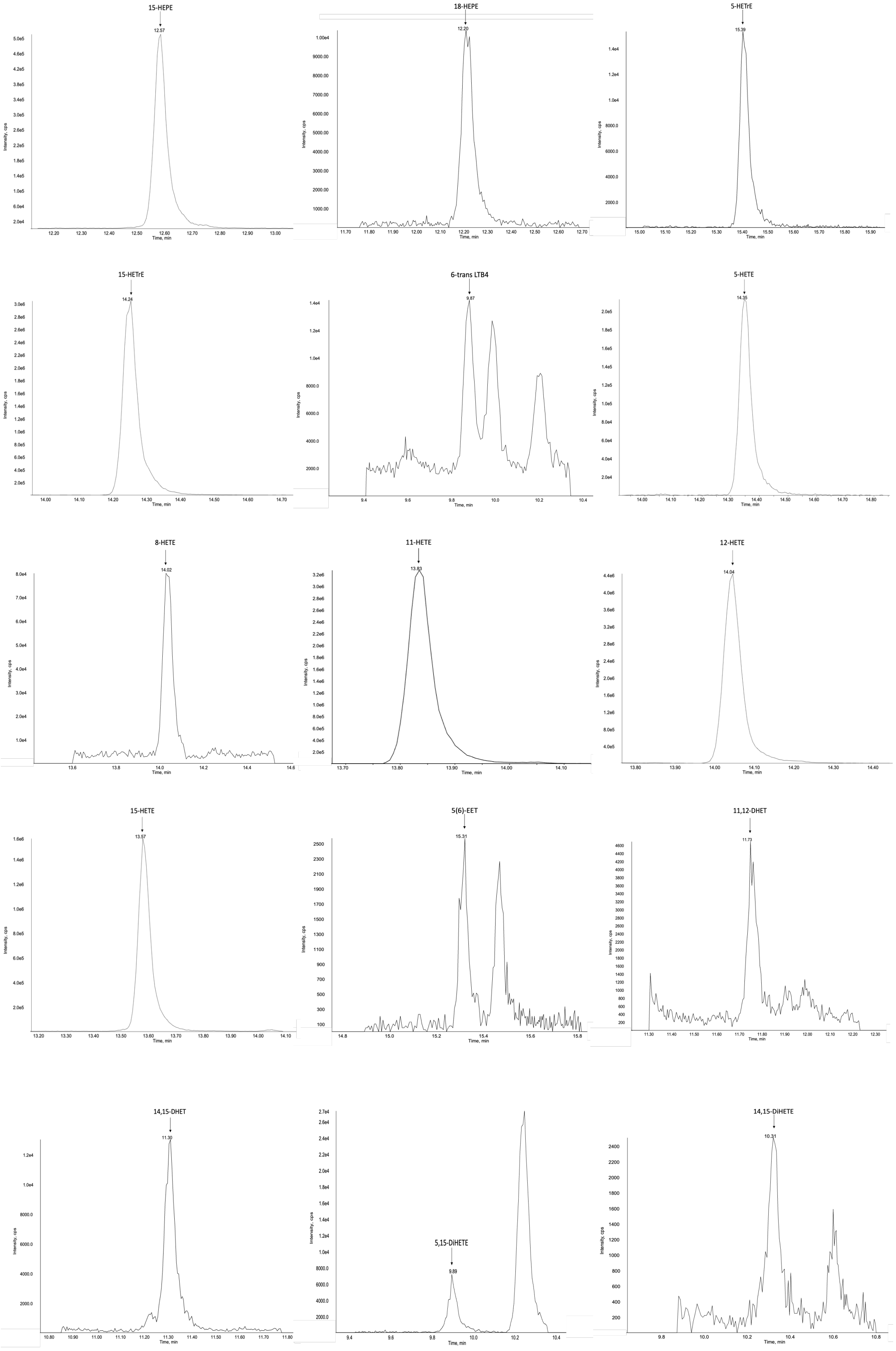
15-HEPE
12.57
18-HEPE
12.20
5-HETrE
15.39
15-HETrE
14.24
6-trans LTB4
9.87
5-HETE
14.35
8-HETE
14.02
11-HETE
13.83
12-HETE
14.04
15-HETE
13.57
5(6)-EET
15.31
11,12-DHET
11.73
14,15-DHET
11.30
5,15-DiHETE
9.89
14,15-DiHETE
10.31
Intensity, cps
Time, min

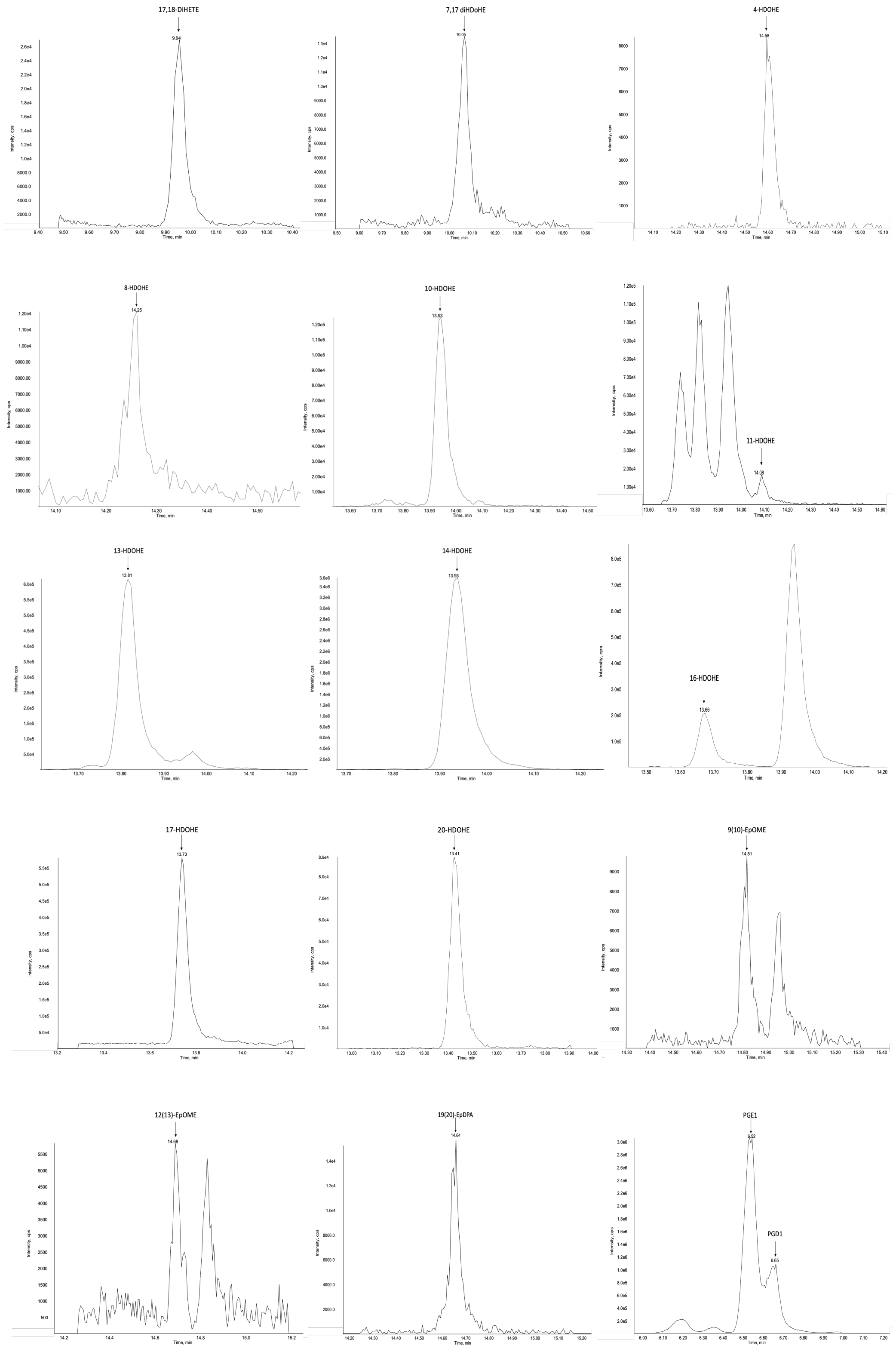

Supplemental Fig. S1. Example LC/MS/MS chromatograms of detected oxylipins

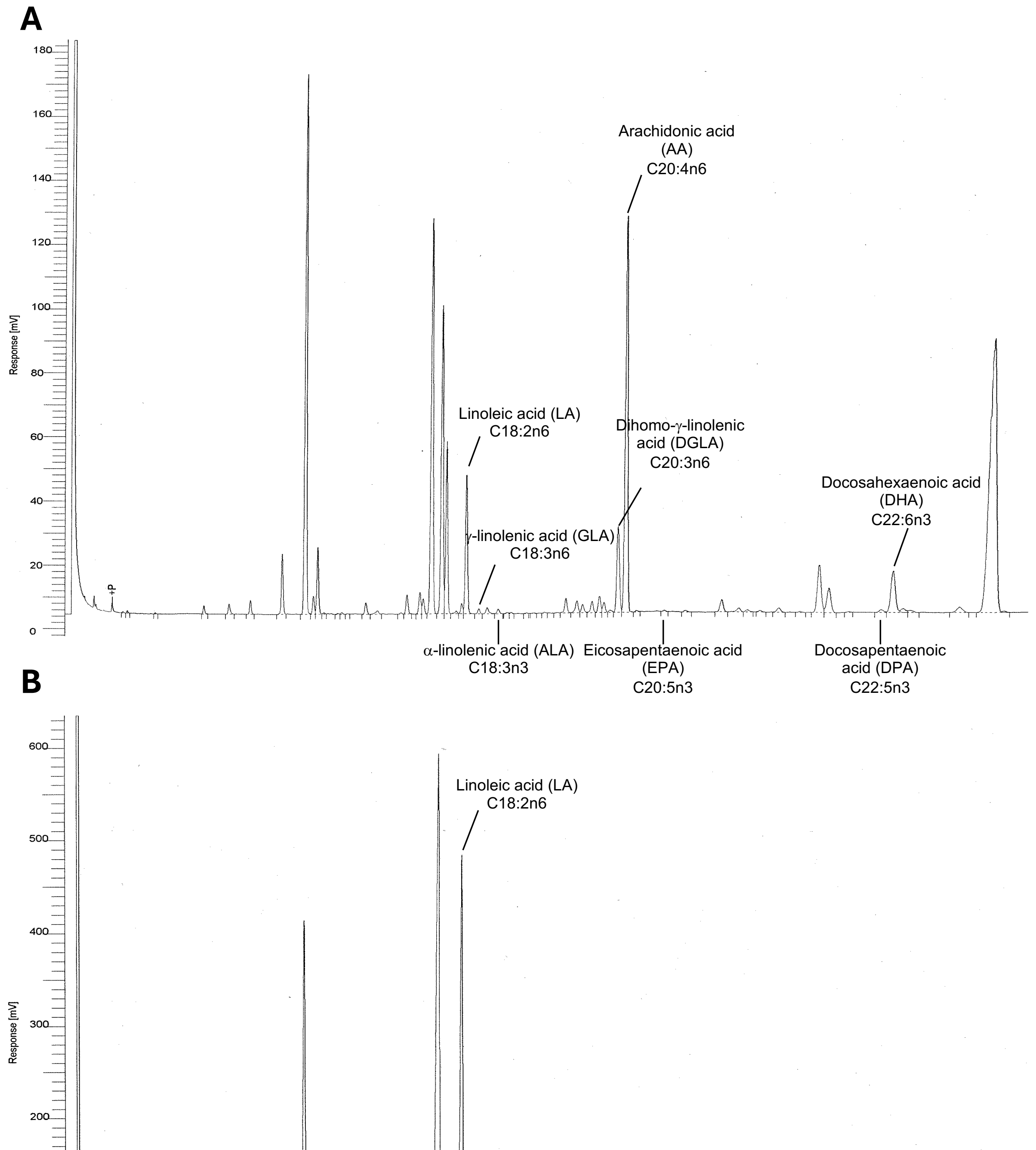

Supplemental Fig. S2. Example GC-FID chromatogram of fatty acids. Shown are fatty acids of interest in the total polar lipid (A) and triacylglycerol fraction (B)

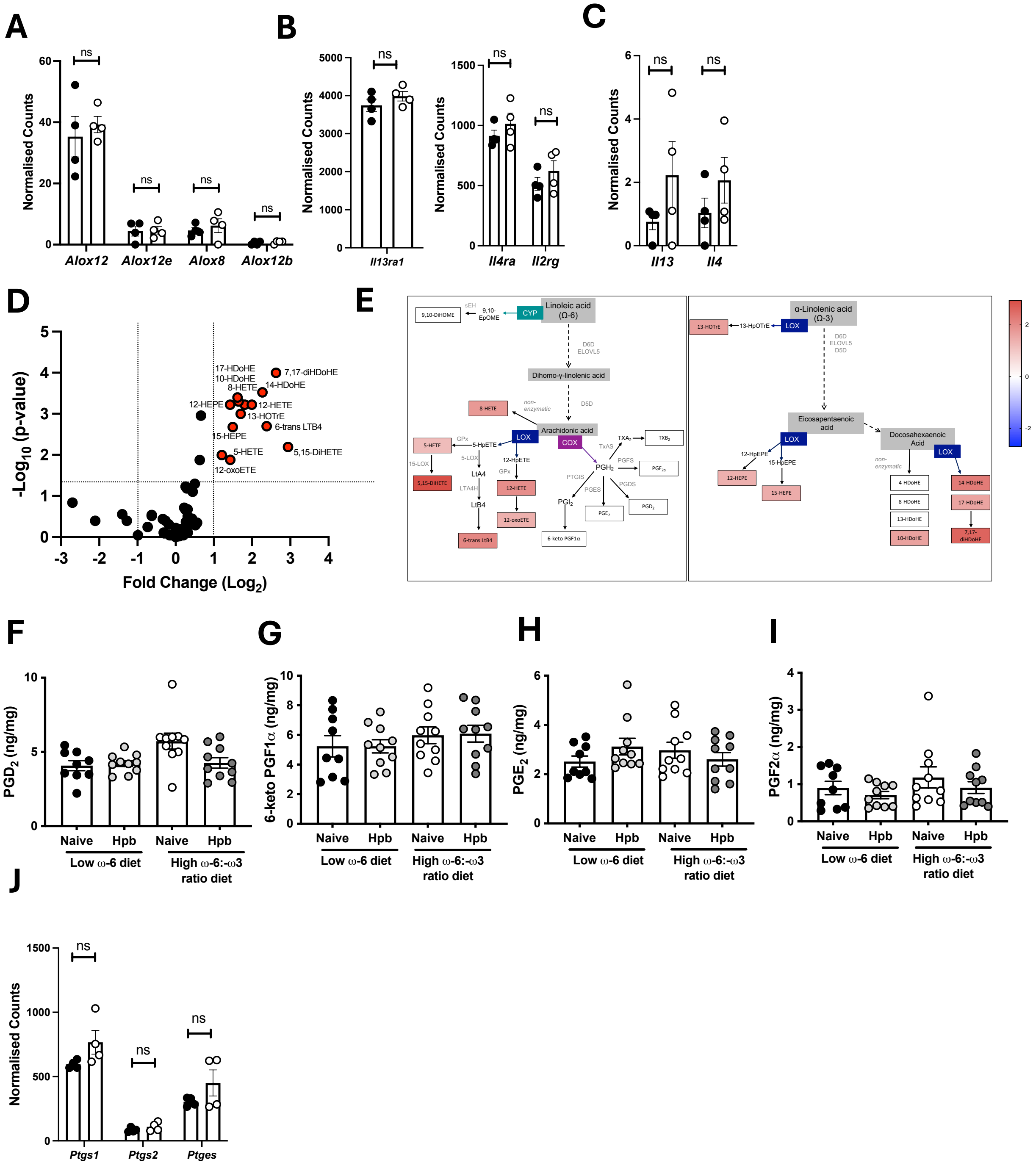

Supplemental Fig. S3. Helminth infection increases production of 12/15-LOX oxylipins, derived from both ω-6 and ω-3 but does not increase COX-derived prostaglandin production, nor the expression of genes involved in prostaglandin synthesis. Naive mice were fed a low ω-6 diet (chow) or a modified AIN-76A diet with a high ω-6:ω-3 ratio than AIN-76A (mAIN-76A) for one week, then

infected with 200 *H. polygyrus* L3 larvae for 14 days (Hpb), or maintained as naïve uninfected controls, before lipidomic or RNAseq analysis of the colon. Expression levels of *Alox12, Alox12e, Alox8, Alox12b* (A), *Il13ra1, Il4ra, Il2rg* (B), *Il13* and *Il4* (C) in the colon of naïve and helminth infected mice fed a high ω-6:ω-3 ratio diet, taken at day 14 post-infection. Volcano plot of all colon oxylipins, highlighting those significantly increased (red symbols) or decreased (blue symbols) in helminth-infected mice fed a low ω-6 diet, compared to naïve uninfected mice fed a low ω-6 diet (D). Simplified pathway representation of the Log_2 fold change of oxylipin for helminth infected mice fed a low ω-6 diet, compared to naïve mice fed a low ω-6 diet as a heatmap scaled from highest amount (dark red, value 2.9) to lowest amount (dark blue, value -2.7) (E). ng per mg amount of the oxylipins PGD_2 (F), 6-keto PGF1α (G), PGE_2 (H) and PGF2α (I). Expression levels of *Ptgs1, Ptgs2* and *Ptges* (J) in the colon of naïve and helminth infected mice fed a high ω-6:ω-3 ratio diet, taken at day 14 post-infection. Experiments shown are pooled data from two separate experiments with n≥4 mice/group (D-I), or one experiment with n=4 mice/group (A-C, J). Unpaired T-test *p<0.05, **p<0.01, ***p<0.001, ****p<0.0001, error bars SEM.

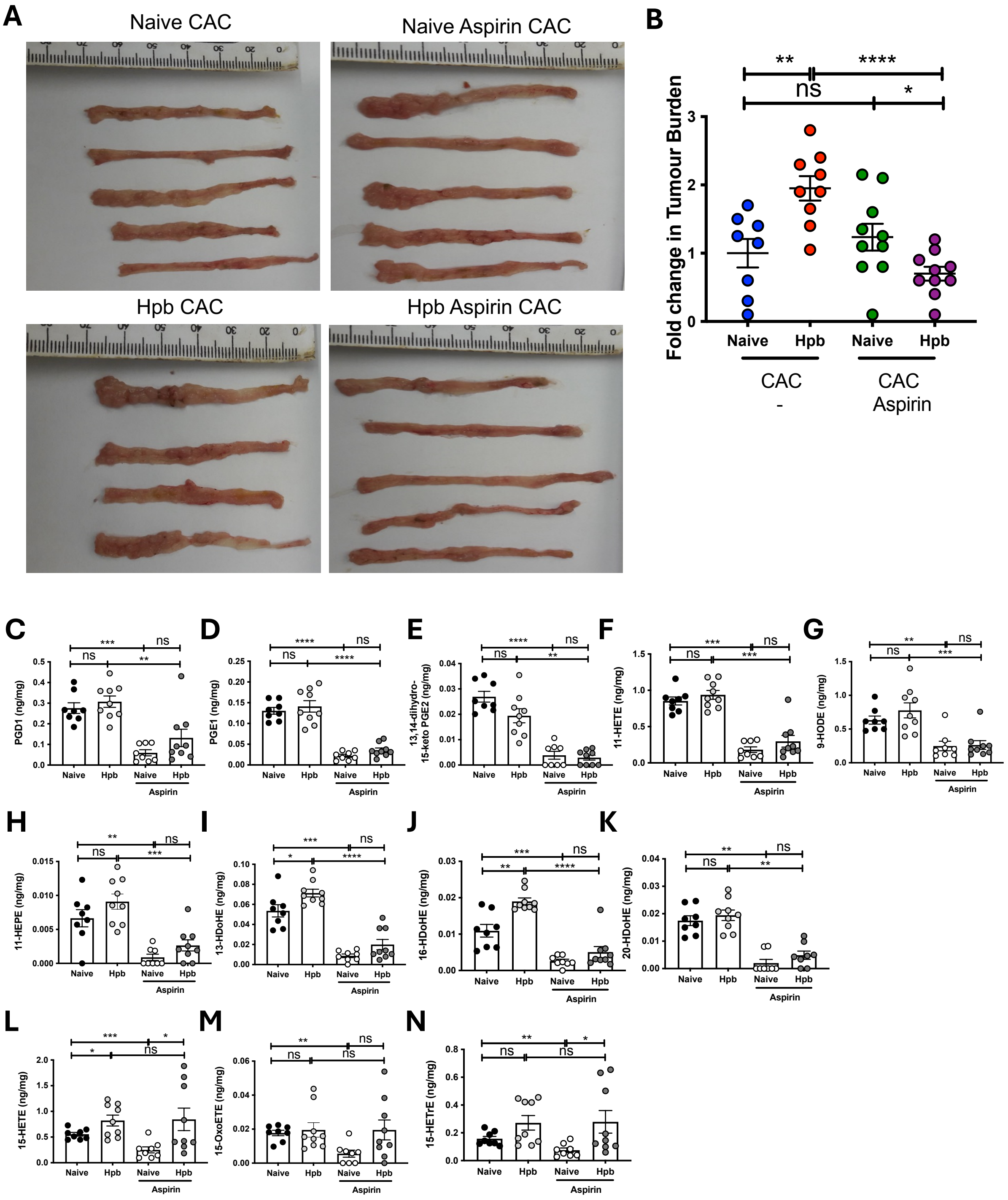

Supplemental Fig. S4. Aspirin treatment significantly reduces CAC and prostaglandin formation in Hpb-infected mice. Mice fed a high ω-6:ω-3 ratio diet were given 25mg/kg/day aspirin in the water from day -15 to day 0 of Hpb infection, or the equivalent time to uninfected (naive) mice. At day 0, lipidomic

analysis was performed, or aspirin-treated mice were placed onto water and CAC was initiated by administering AOM at day 0, followed by three fortnightly cycles of DSS in the water. Representative images of tumour formation in the colon at day 59 following administration of AOM (A). Fold change in tumour burden in treatment groups, compared to the mean of naïve CAC control burden (B). ng per mg amount of the oxylipins PGD1 (C), PGE1 (D), 13,14-dihydro-15-keto-PGE_2 (E), 11-HETE (F), 9-HODE (G), 11-HEPE (H), 13-HDoHE (I), 16-HDoHE (J), 20-HDoHE (K), 15-HETE (L), 15-OxoETE (M) and 15-HETrE (N). Experiments shown are one representative of two experiments with n≥4 mice/group (A) or pooled from two experiments with n≥4 mice/group (B-N). Unpaired T-test *p<0.05, **p<0.01, ***p<0.001, ****p<0.0001, error bars SEM.

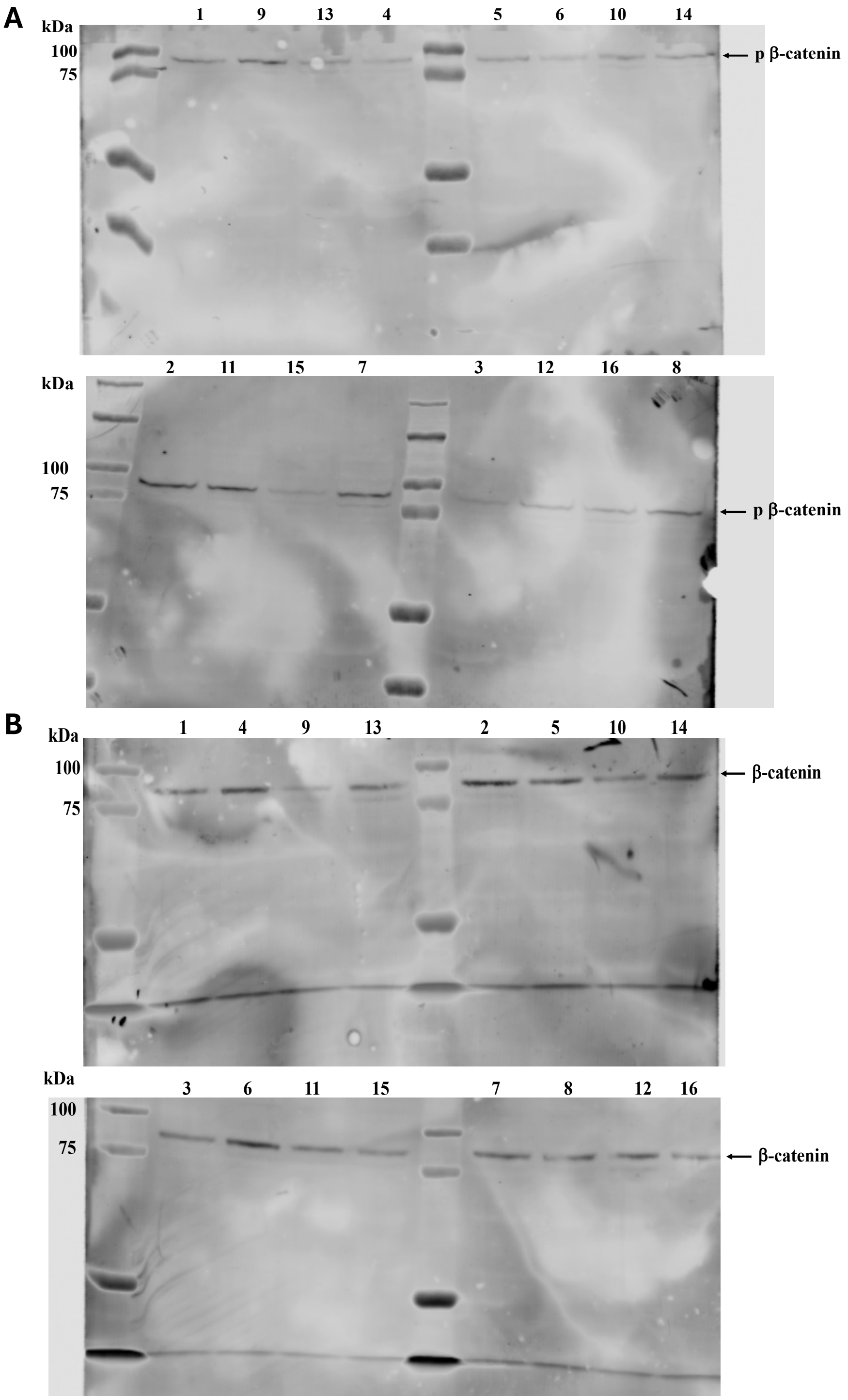

Supplemental Fig. S5. Western blots of p-β-catenin Ser^{552} (A) and β-catenin (B) (both 92kDa). Original western blots of colon samples (summarised in **Figure 6C**). Shown are Naive vehicle (1-3), Naive vehicle AOM/DSS (4-8), Hpb vehicle AOM/DSS (9-12), Hpb EP2/EP4 AOM/DSS (13-16).

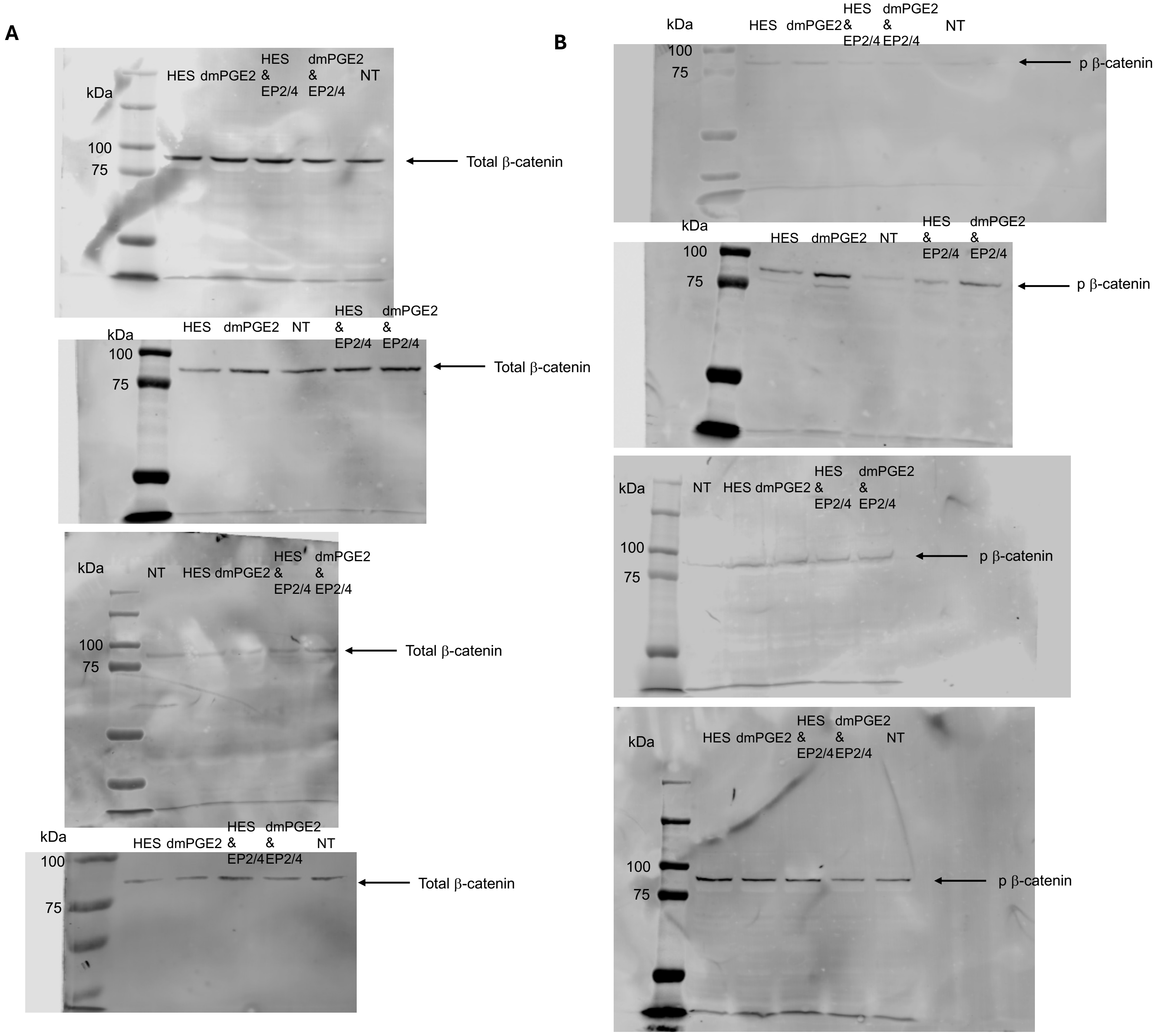

Supplemental Fig. S6. Western blots of β-catenin (A) and p-β-catenin Ser^{552} (B) (both 92kDa). Original western blots of CMT-93 cell cultures (summarised in **Figure 6H, I**). Shown are treatment with dmPGE2, dmPGE2 & EP2/EP4 antagonists, Hpb excretory/secretory product (HES), HES & EP2/EP4 antagonists or cells with vehicle control (NT).

Supplemental Table S1. Multiple reaction monitoring (MRM) Transitions

Q1 MASS	Q3 MASS	R.T	I.D	DP	CE	CXP
319.2	115.1	14.4	5-HETE	-55	-19	-7
319.2	155.101	14.1	8-HETE	-65	-18	-8
319.2	167.1	14.27	9-HETE	-50	-20	-9
319.2	167.102	13.91	11-HETE	-60	-19	-9
319.2	179.1	14.11	12-HETE	-65	-18	-12
319.2	219.1	13.65	15-HETE	-55	-18	-14
319.2	275.1	12.64	20-HETE	-85	-21	-11
317.2	115.1	13.17	5-HEPE	-60	-20	-10
317.2	155.1	12.8	8-HEPE	-65	-19	-8
317.2	167.1	12.99	9-HEPE	-50	-18	-12
317.2	167.101	12.69	11-HEPE	-50	-20	-13
317.2	179.1	12.91	12-HEPE	-65	-18	-8
317.2	219.1	12.63	15-HEPE	-65	-16	-10
317.2	259.1	12.25	18-HEPE	-50	-15	-11
343.2	101.1	14.66	4-HDoHE	-50	-17	-9
343.2	141.1	14.2	7-HDoHE	-50	-21	-9
343.2	189.1	14.31	8-HDoHE	-50	-19	-9
343.2	153.101	13.99	10-HDoHE	-55	-21	-5
343.2	121.1	14.14	11-HDoHE	-60	-18	-10
343.2	193.1	13.87	13-HDoHE	-55	-19	-9
343.2	205.1	13.99	14-HDoHE	-45	-17	-9
343.2	233.101	13.73	16-HDoHE	-55	-17	-10
343.2	201.1	13.79	17-HDoHE	-70	-15	-10
343.2	241.101	13.47	20-HDoHE	-55	-17	-11
295.2	171.1	13.34	9-HODE	-85	-23	-9
295.2	195.1	13.28	13-HODE	-85	-23	-7
293.2	171.1	12	9-HOTrE	-60	-20	-8
293.2	195.101	12.2	13-HOTrE	-70	-22	-12
321.2	115.1	15.49	5-HETrE	-70	-19	-9
321.2	221.1	14.29	15-HETrE	-70	-21	-11
293.2	185.1	14	9-OxoODE	-85	-23	-13
293.2	195.1	13.72	13-OxoODE	-85	-25	-12
317.2	273.1	15.06	5-OxoETE	-65	-20	-11
317.2	153.1	14.36	12-OxoETE	-75	-20	-10
317.2	113.1	14	15-OxoETE	-60	-22	-8
313.2	201.1	10.9	9,10-DiHOME	-80	-29	-8
313.2	183.1	10.62	12,13-DiHOME	-80	-28	-12
337.2	145.1	12.64	5,6-DiHETrE	-75	-24	-10

337.2	127.1	12.14	8,9-DiHETrE	-70	-25	-8
337.2	167.1	11.79	11,12-DiHETrE	-65	-26	-8
337.2	207.1	11.45	14,15-DiHETrE	-65	-25	-10
335.2	115.1	11.2	5,6-DiHETE	-60	-23	-8
335.2	115.101	9.92	5,15-DiHETE	-60	-21	-9
335.2	235.1	9.63	8,15-DiHETE	-65	-22	-4
335.201	207.1	10.35	14,15-DiHETE	-65	-23	-10
335.2	247.1	9.97	17,18-DiHETE	-65	-24	-8
349.2	195.1	3.21	RvE1	-65	-22	-10
375.2	215.1	7.47	RvD1	-55	-23	-9
375.2	141.1	6.8	RvD2	-65	-21	-11
375.2	147.1	6.49	RvD3	-65	-24	-12
359.2	199.1	10.09	7,17-diHDoHE	-65	-22	-17
337.2	195.1	11.5	LTB3	-65	-22	-8
335.2	195.1	10.22	LTB4	-70	-23	-11
365.2	347.2	3.24	20-carboxy LTB4	-80	-25	-8
351.2	195.1	3.55	20-hydroxy LTB4	-80	-25	-8
335.2	195.101	9.89	6-trans LTB4	-65	-23	-9
351.2	115.1	7.32	LXA4	-55	-19	-10
359.2	250.1	10.1	Mar 1	-60	-23	-11
361.2	263.1	10.38	7,17-diHDPA	-65	-20	-4
295.2	171.101	14.86	9(10)-EpOME	-80	-21	-10
295.2	195.101	14.74	12(13)-EpOME	-80	-19	-8
319.2	191.1	15.37	5(6)-EET	-60	-16	-7
319.2	167.101	15.15	8(9)-EET	-60	-15	-7
319.2	167.103	15.15	11(12)-EET	-60	-18	-8
319.2	219.101	14.84	14(15)-EET	-65	-18	-6
317.2	127.1	14.2	8(9)-EpETE	-70	-18	-8
317.2	167.102	14.12	11(12)-EpETE	-70	-15	-11
317.2	207.1	14.04	14(15)-EpETE	-70	-18	-6
317.2	215.1	13.7	17(18)-EpETE	-75	-16	-10
343.2	113.1	15.2	7(8)-EpDPA	-60	-16	-7
343.2	153.1	15.08	10(11)-EpDPA	-65	-15	-7
343.2	193.101	15.02	13(14)-EpDPA	-70	-15	-7
343.2	233.1	14.97	16(17)-EpDPA	-55	-16	-9
343.2	241.1	14.71	19(20)-EpDPA	-70	-18	-11
353.2	317.202	6.65	PGD1	-55	-16	-8
351.2	271.102	6.61	PGD2	-50	-22	-8
349.2	269.101	5.26	PGD3	-50	-17	-11
353.2	317.2	6.53	PGE1	-60	-18	-10
351.2	271.1	6.2	PGE2	-60	-19	-12

349.2	269.1	4.86	PGE3	-60	-17	-10
333.2	175.1	8.82	PGB2	-60	-24	-10
351.2	235.1	7.33	13,14-dihydro-15-keto PGE2	-55	-19	-13
351.2	207.1	8.16	13,14-dihydro-15-keto PGD2	-50	-25	-13
353.201	113.101	7.43	13,14-dihydro-15-keto PF2α	-55	-23	-11
351.201	271.101	6.38	11β-PGE2	-55	-23	-7
367.2	143.1	3.22	6-keto PGE1	-55	-23	-9
351.201	271.103	5.94	8-iso PGE2	-55	-21	-10
315.2	271.1	12.44	15-deoxy-Δ12,14-PGJ2	-65	-18	-8
351.2	289.1	5.37	8-iso-15-keto PGF2α	-50	-23	-12
353.2	309.2	5.89	PGF2α	-85	-24	-9
369.201	163.1	3.3	6-keto PGF1α	-75	-26	-10
369.2	169.1	4.83	Thromboxane B2	-60	-22	-12
367.2	305.2	6.24	11-dehydro Thromboxane B2	-60	-20	-10
299.2	198.1	13.22	13(S)-HODE-d4	-60	-25	-7
327.2	116.1	14.32	5(S)-HETE-d8	-55	-19	-8
327.2	184.1	14.02	12(S)-HETE-d8	-60	-20	-12
327.2	226.1	13.55	15(S)-HETE-d8	-65	-22	-11
325.2	281.1	12.6	20-HETE-d6	-70	-21	-8
339.2	197.1	10.17	Leukotriene B4-d4	-65	-21	-9
380.2	141.1	7.41	Resolvin D1-d5	-75	-18	-11
355.2	275.101	6.16	Prostaglandin E2-d4	-60	-23	-12
355.2	275.100	6.58	Prostaglandin D2-d4	-55	-23	-10
357.2	313.2	5.86	Prostaglandin F2α-d4	-80	-24	-9
373.2	173.1	4.79	Thromboxane B2-d4	-55	-22	-10
371.2	309.2	6.21	11-dehydro Thromboxane B2-d4	-55	-21	-11
331.2	167.1	15.09	11(12)-EET -d11	-65	-18	-11

Supplemental Table S2. RNA sequencing (RNA-seq)

Sample ID	Total number of sequenced reads	Total number of uniquely mapped reads	RNA integrity number (RIN)	Ratio of all reads aligned to rRNA regions to total uniquely mapped reads (rRNA rate)	Ratio of exon-mapped reads to total uniquely mapped reads (Expression Profile Efficiency)	Total number of detected transcripts with reads ≥1
Naive 1	69,406,608	59,455,532	8.8	85.66%	92.67%	21,574
Naive 2	67,946,450	58,746,877	8.4	86.46%	92.38%	21,603
Naive 3	63,323,234	55,219,077	9.5	87.2%	92.48%	21,558
Naive 5	61,657,934	52,441,493	9	85.05%	92.3%	23,003
Hpb 1	73,350,508	64,509,544	9.4	87.95%	91.98%	22,686
Hpb 2	66,997,866	57,456,581	8.6	85.76%	92.76%	21,993
Hpb 3	64,736,944	56,181,780	9.4	86.78%	91.6%	23,162
Hpb 5	72,545,494	62,846,757	8.8	86.63%	91.68%	21,887

Mus musculus BALB_cJ_v1 genome available from Ensemble (EMBL-EBI) (GCA_001632525.1)

Supplemental Table S3. qRT-PCR primer sequences

**Gene**	**Sequence**
Gapdh	FOR: 5'-ATGACATCATCAAGAAGG TGGTG-3'
	REV: 5'- CATACCAGGAAATGAGCTTG-3'
Alox5	FOR: 5'-TGTCTGAGGTGTTTGGTATCG-3'
	REV: 5'-AAGGCCATACTCGCAGATAAG-3'
Alox15	FOR: 5'-CTGAAGCGGTCTACTTGTCTC-3'
	REV: 5'-ACATTGGCCTTGATCCCATC-3'
